# Supplementary material for: Pharmaceutical targeting of OTUB2 sensitizes tumors to cytotoxic T cells via degradation of PD-L1
Source: Nat Commun. 2024 Jan 2;15:9. doi: 10.1038/s41467-023-44466-7 (PMC10761827; doi:10.1038/s41467-023-44466-7)

# Supporting Information

# Pharmaceutical targeting of OTUB2 sensitizes tumors to cytotoxic T cells via degradation of PD-L1

Wenfeng Ren[1†], Zilong Xu[1†], Yating Chang[1,2†], Fei Ju[1†], Hongning Wu[1,2†], Zhiqi Liang[1,2†], Min Zhao[1], Naizhen Wang[1], Yanhua Lin[1], Chenhang Xu[1,2], Shengming Chen[1], Yipeng Rao[1], Chaolong Lin[1,3], Jianxin Yang[4,5], Pingguo Liu[4,5*], Jun Zhang[1,3*], Chenghao Huang[1,3*], Ningshao Xia[1,2,3*]

[1]State Key Laboratory of Molecular Vaccinology and Molecular Diagnostics, National Institute of Diagnostics and Vaccine Development in Infectious Diseases, Department of Laboratory Medicine, School of Public Health, Xiamen University, Xiamen, Fujian, China, 361102

[2]School of Life Sciences, Xiamen University, Xiamen, Fujian, China, 361102

[3]Xiang An Biomedicine Laboratory, Xiamen, Fujian China, 361102

[4]Department of Hepatobiliary & Pancreatic Surgery, The National Key Clinical Specialty, Zhongshan Hospital of Xiamen University, School of Medicine, Xiamen University, Xiamen, Fujian, China, 361004

[5]Fujian Provincial Key Laboratory and Chronic Liver Disease and Hepatocellular Carcinoma, Xiamen, Fujian, China, 361004

†These authors contributed equally: Wenfeng Ren, Zilong Xu, Yating Chang, Fei Ju, Hongning Wu and Zhiqi Liang.

*Corresponding authors: nsxia@xmu.edu.cn; huangchenghao@xmu.edu.cn; zhangj@xmu.edu.cn; pgliu@xmu.edu.cn.

# Inventory of Supporting Information

Supplementary Fig. 1. (Related to Fig. 1)

Supplementary Fig. 2. (Related to Fig. 1)

Supplementary Fig. 3. (Related to Fig. 1)

Supplementary Fig. 4. (Related to Fig. 1)

Supplementary Fig. 5. (Related to Fig. 1)

Supplementary Fig. 6. (Related to Fig. 1)

Supplementary Fig. 7. (Related to Fig. 2)

Supplementary Fig. 8. (Related to Fig. 3)

Supplementary Fig. 9. (Related to Fig. 3)

Supplementary Fig. 10. (Related to Fig. 3)

Supplementary Fig. 11. (Related to Fig. 3)

Supplementary Fig. 12. (Related to Fig. 4)

Supplementary Fig. 13. (Related to Fig. 5)

Supplementary Fig. 14. (Related to Fig. 6)

Supplementary Fig. 15. (Related to Fig. 6)

Supplementary Fig. 16. (Related to Fig. 7)

Supplementary Fig. 17. (Related to Fig. 8)

Supplementary Fig. 18. (Related to Fig. 8)

Supplementary Fig. 19. (Related to Fig. 9)

Supplementary Fig. 20. (Related to Fig. 9)

Supplementary Fig. 21. (Related to Fig. 9)

Supplementary Fig. 22. (Related to Fig. 9)

Supplementary Fig. 23. (Related to Fig. 9)

Supplementary Fig. 24. (Related to Fig. 9)

Supplementary Fig. 25. (Related to Fig. 9)

Supplementary Fig. 26. (Related to Fig. 9)

Supplementary Fig. 27. (Related to Fig. 9)

Supplementary Fig. 28. (Related to Fig. 9)

Supplementary Fig. 29.

Supplementary Table. 1.

Supplementary Table. 2.

Supplementary Table. 3.

Supplementary Table. 4.

Supplementary Table. 5.

Supplementary Table. 6.

Supplementary Table. 7.

Source Data.

## Supplementary Fig. 1

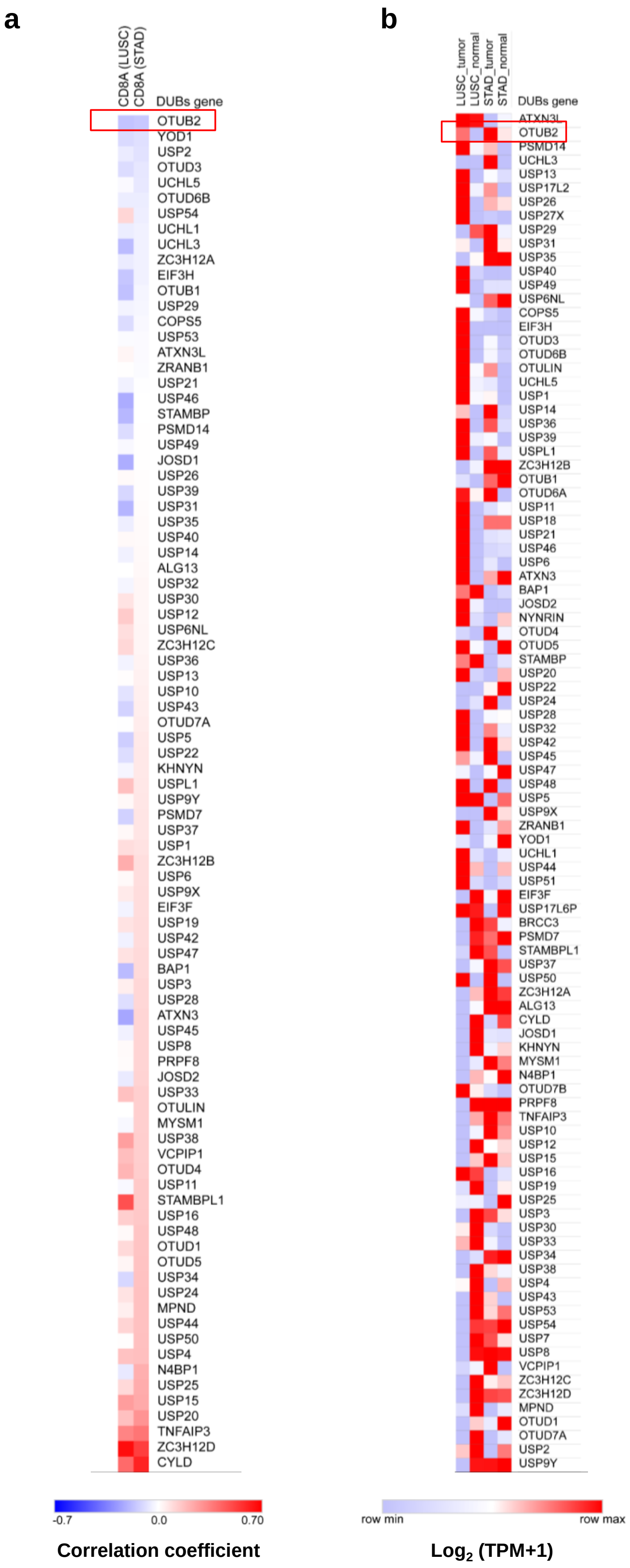

**Supplementary Fig. 1. Bioinformatic analyses identifies OTUB2 as a negative regulator in the TME. a,** Pearson's correlation coefficients between *CD8A* and 103 DUBs using the indicated datasets from TCGA database. **b,** Heatmap visualization of the gene expression levels of OTUB2 in different cancer tissues (Tumor) and adjacent paracancerous tissues (Normal) in the indicated datasets from TCGA database. Data are shown as $\log_2$ (TPM+1). TPM, transcripts per million. Expression data of USP52, HAUSP, PPPDE1 and CSN6 are not available in the indicated datasets.

## Supplementary Fig. 2

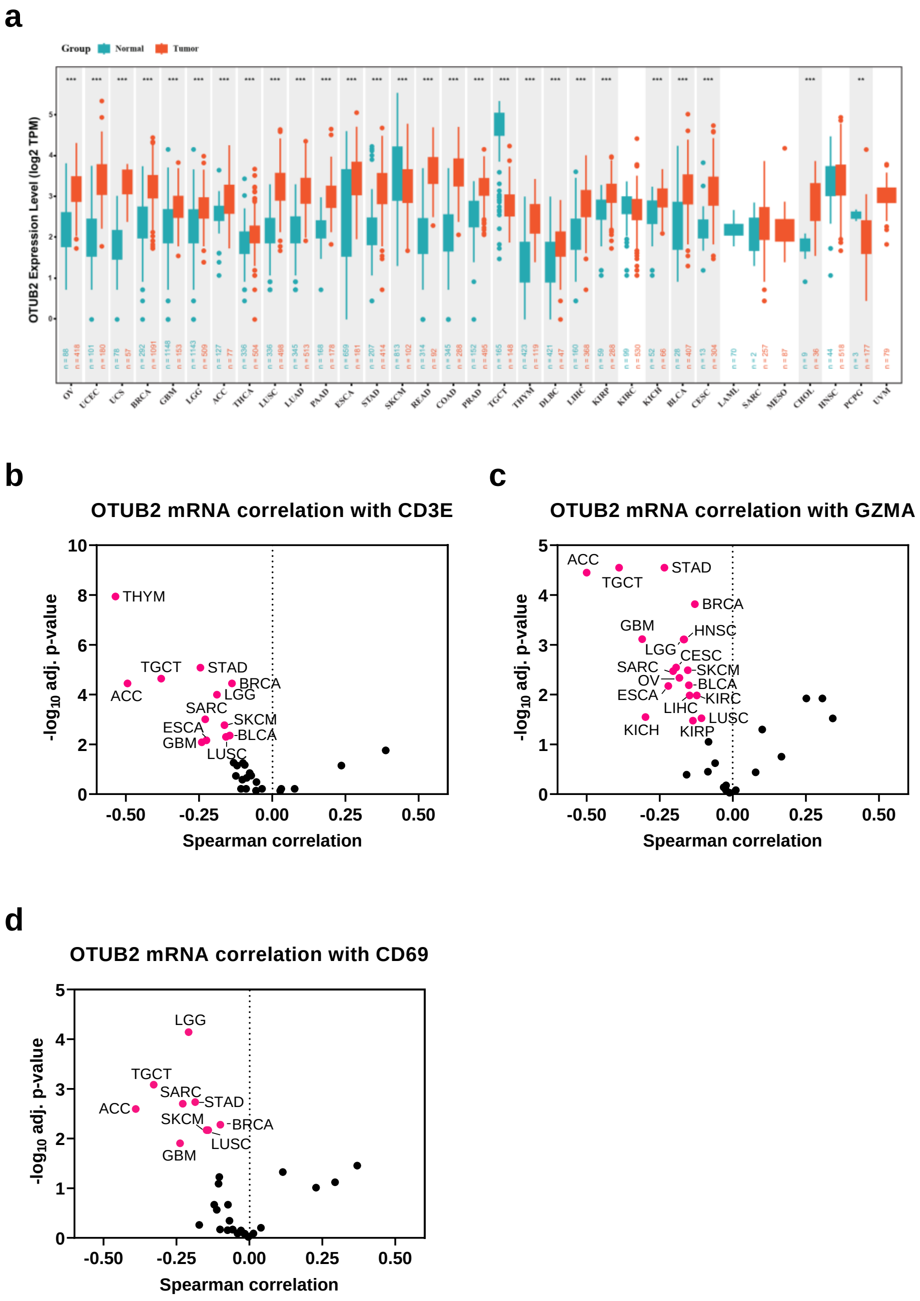

**Supplementary Fig. 2. Pancancer dissection of the transcriptomic signature of $CD8^+$ T-cell-associated genes associated with OTUB2 expression in the TCGA database. a,** Differential expression of *OTUB2* across tumor samples for all cancer types compared with normal samples. Analyses were performed using data from TCGA datasets. Cancer types with significant upregulation of *OTUB2* are labeled in gray. **b,** Volcano plot of the Spearman correlation between *OTUB2* and *CD3E* mRNA expression in 31 cancer types. Data from the TCGA database were analyzed. Magenta points indicate cancer types in which *OTUB2* was significantly negatively correlated with *CD3E* (adjusted $P < 0.05$). **c,** Volcano plot of the Spearman correlation between *OTUB2* and *GZMA* mRNA expression in 32 cancer types. Data from the TCGA database were analyzed. Magenta points indicate cancer types in which *OTUB2* was significantly negatively correlated with *GZMA* (adjusted $P < 0.05$). **d,** Volcano plot of the Spearman correlation between *OTUB2* and *CD69* mRNA expression in 32 cancer types. Magenta points indicate cancer types in which *OTUB2* was significantly negatively correlated with *CD69* (adjusted $P < 0.05$). The data are presented as the mean ± s.e.m., and P values were calculated using the Spearman's correlation test (**b**-**d**) except for in **a,** in which P values were calculated by an unpaired two-sided Student's t test. $^{**}P < 0.01$; $^{***}P < 0.001$.

# Supplementary Fig. 3

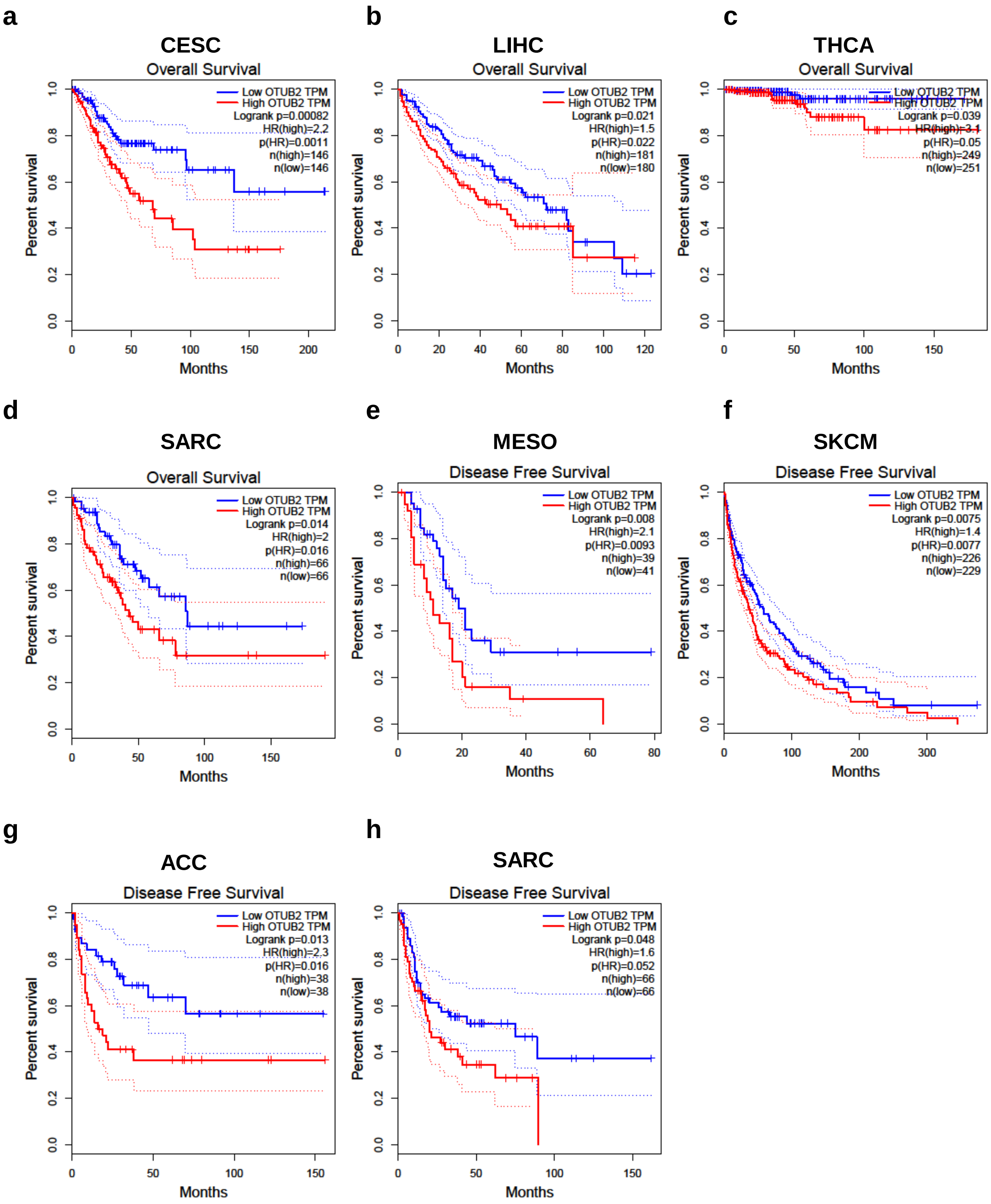

**Supplementary Fig. 3. Associations between OTUB2 expression and the survival of patients in the TCGA database. a-d,** Overall survival of CESC patients (**a**), LIHC patients (**b**), THCA patients (**c**) and SARC patients (**d**) grouped based on OTUB2 expression; data from the TCGA database were analyzed. **e-h,** Disease-free survival of MESO patients (**e**), SKCM patients (**f**), ACC patients (**g**) and SARC patients (**h**) grouped based on OTUB2 expression; data from the TCGA database were analyzed. The data are presented as the mean ± s.e.m., and P values were calculated by the log-rank test (**a**-**h**).

**Supplementary Fig. 4**

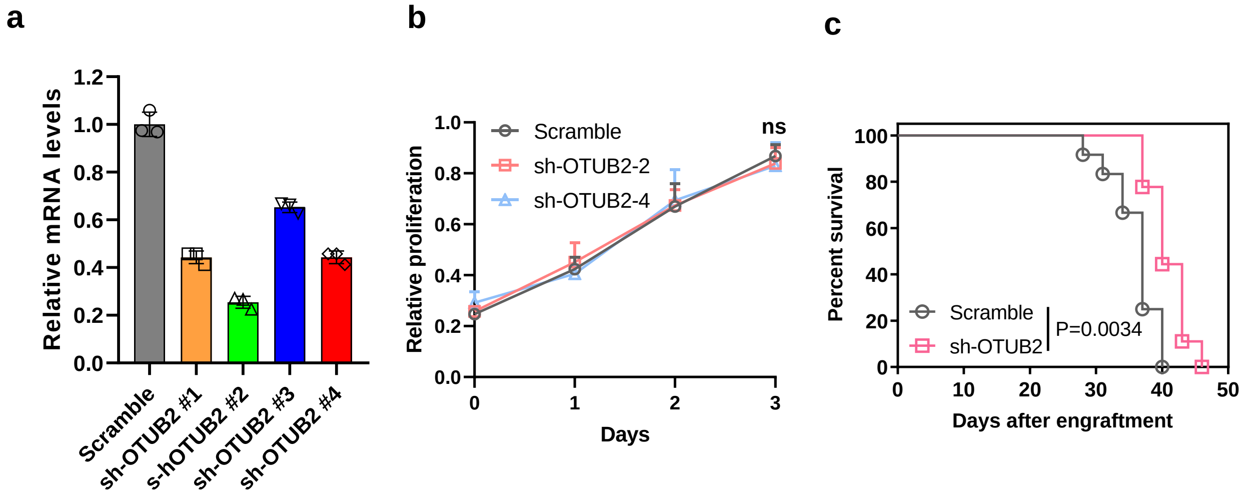

**Supplementary Fig. 4. Effects of OTUB2 KD on MC38 tumor-cell growth. a,** Knockdown (KD) efficiency of four shRNAs targeting mouse OTUB2. OTUB2 mRNA levels in MC38 tumor cells expressing control shRNA (Scramble) or OTUB2-specific shRNAs (shRNA #1-#4). n = 3 biologically independent experiments. **b,** Vector control (scramble) or OTUB2-KD (sh-OTUB2) MC38 tumor cells were seeded and cell growth was examined using a proliferation assay. n = 3 biologically independent experiments. **c,** Kaplan–Meier survival analysis of host animals bearing vector control or OTUB2-KD MC38 tumors. n = 5 mice per group. The data are presented as the mean ± s.e.m. from at least two independent experiments, and P values were determined by an unpaired two-sided Student's t test (**b**), except for in **c,** in which P values were determined by the log-rank test. ns, not significant.

## Supplementary Fig. 5

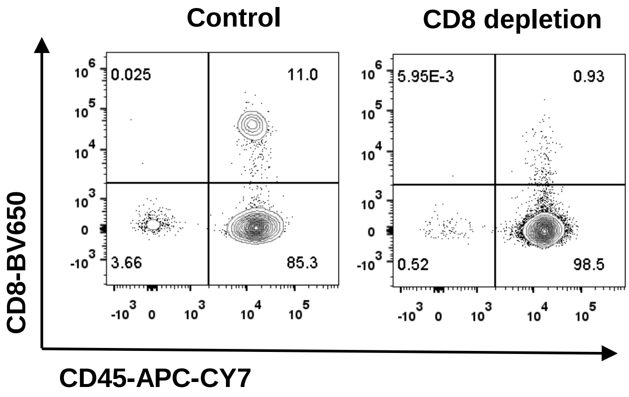

**Supplementary Fig. 5. Depletion of $CD8^+$ T cells *in vivo*.** Representative plots of $CD8^+$ T-cell depletion from 1 of 3 independent experiments are shown.

## Supplementary Fig. 6

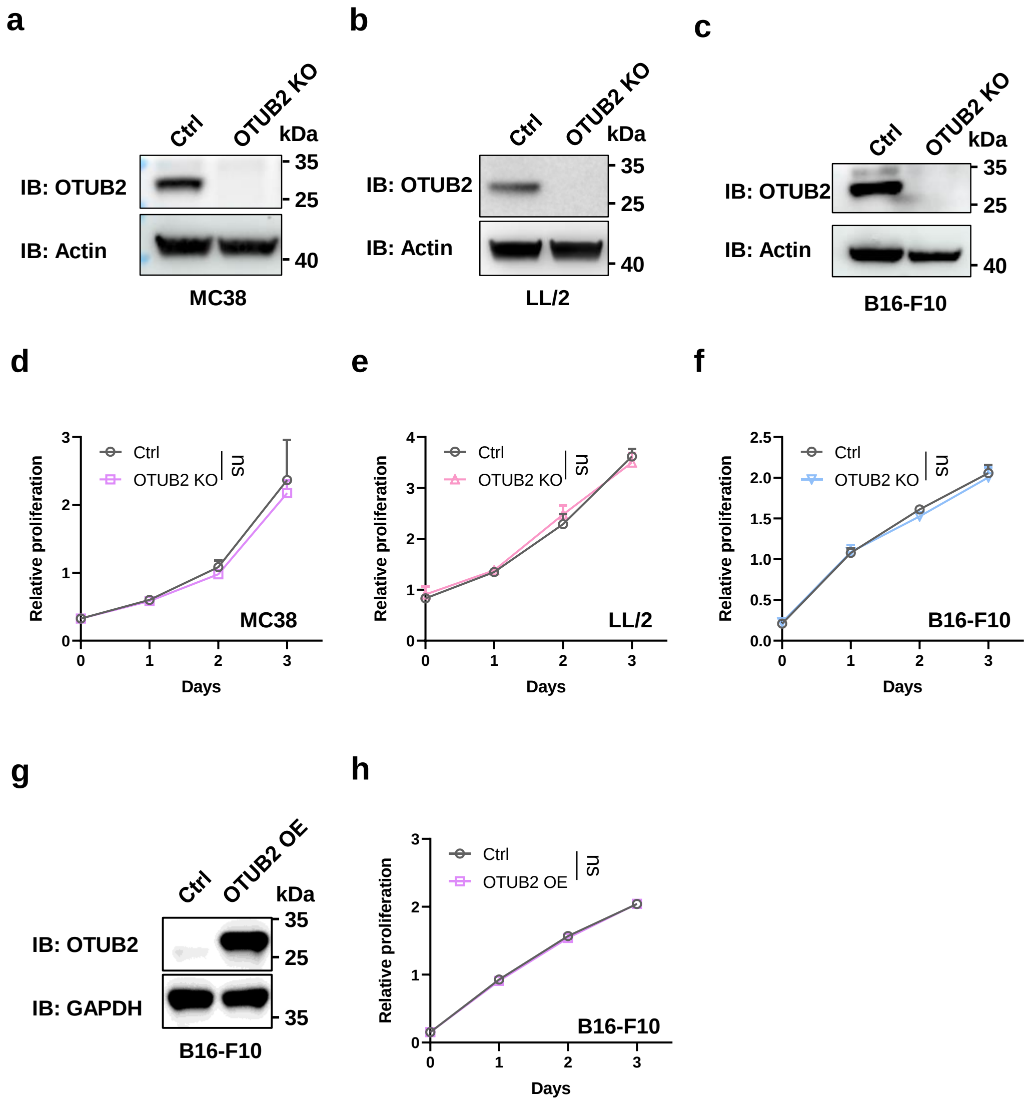

**Supplementary Fig. 6. Effects of OTUB2 KO or OE on tumor-cell growth *in vitro*.** **a-c,** Western blot analysis of the expression of OTUB2 in murine tumor cell lines (MC38, LL/2 and B16-F10) with or without OTUB2 knockout (OTUB2-KO). Actin was used as a protein loading control. **d-f,** Cell growth of control or OUTB2-KO murine tumor cells (MC38, LL/2 and B16-F10). **g,** Western blot analysis of the expression of OTUB2 in B16-F10 tumor cell lines overexpressing OTUB2 (OTUB2-OE). GAPDH was used as a protein loading control. **h,** Cell growth of control or OUTB2-OE B16-F10 tumor cells. The data are presented as the mean ± s.e.m. from three independent experiments (n = 3), and P values were calculated using two-way analysis of variance (ANOVA). Source data are provided as a Source Data file. ns, not significant.

## Supplementary Fig. 7

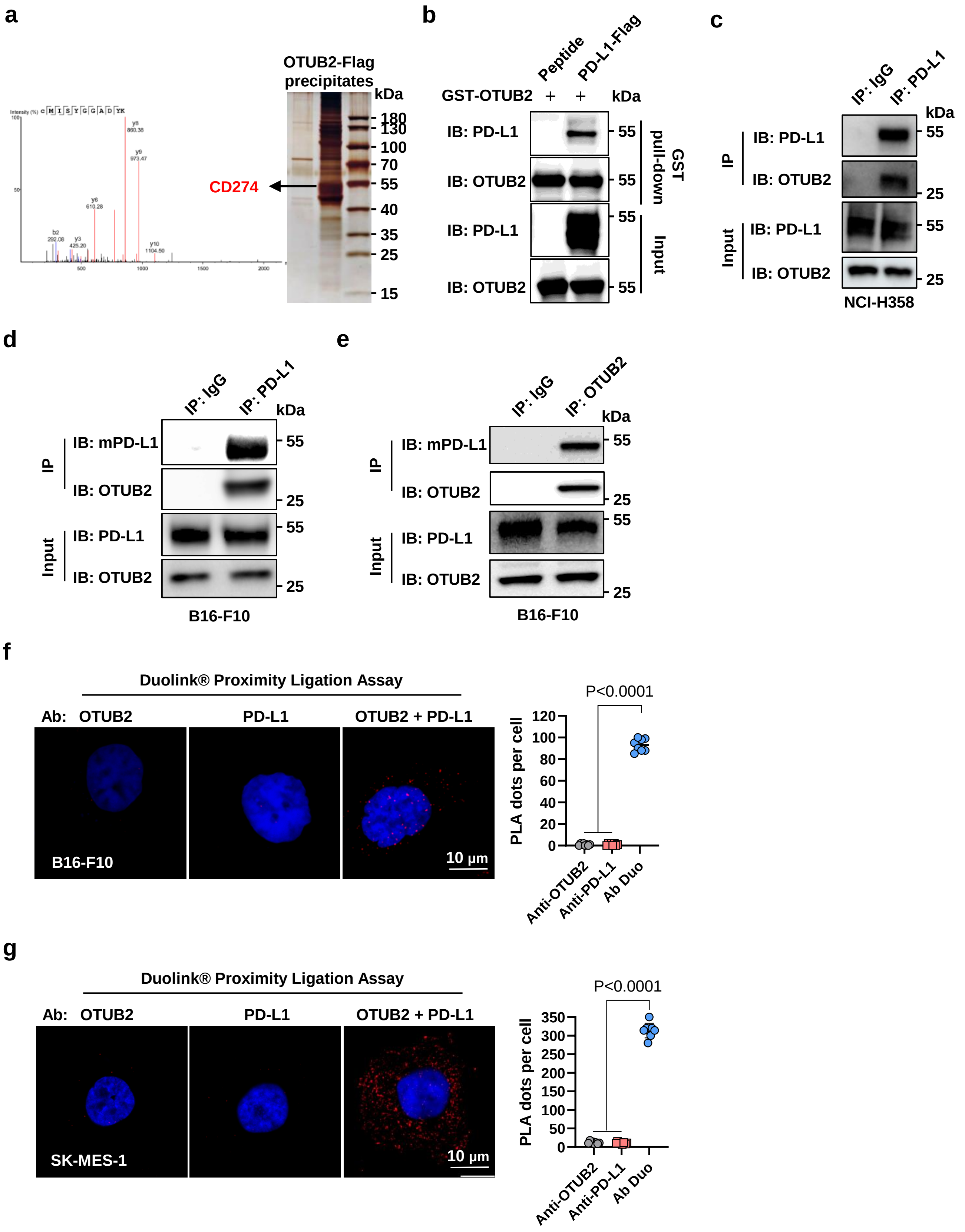

**Supplementary Fig. 7. OTUB2 interacts with PD-L1 in B16-F10 and SK-MES-1 cells. a,** Silver staining of OTUB2-Flag precipitates from B16-F10 cells. **b,** The *in vitro* interaction between purified PD-L1-Flag proteins and GST-OTUB2 proteins examined by *in vitro* GST pull-down assays. **c,** Immunoprecipitation was performed with an anti-PD-L1 antibody to examine the interaction between endogenous OTUB2 and PD-L1 in NCI-H358 cells. **d,** Immunoprecipitation was performed with an anti-PD-L1 antibody to examine the interaction between endogenous OTUB2 and PD-L1 in B16-F10 cells. **e,** Immunoprecipitation was performed with an anti-OTUB2 antibody to examine the interaction between endogenous OTUB2 and PD-L1 in B16-F10 cells. **f,g,** *In situ* interaction between OTUB2 and PD-L1 in B16-F10 (**f**) and SK-MES-1 (**g**) cells. After fixation, a Duolink *in situ* PLA was performed with anti-OTUB2 and anti-PD-L1 antibodies to assess the OTUB2-PD-L1 interaction. Quantification of the PLA dots indicating PD-L1-OTUB2 interactions is shown as the mean ± s.e.m.. Scale bar, 10 μm. n = 8 biologically independent samples. The data are presented as the mean ± s.e.m., and P values were calculated using an unpaired two-sided Student's t test (**f** and **g**). Source data are provided as a Source Data file.

## Supplementary Fig. 8

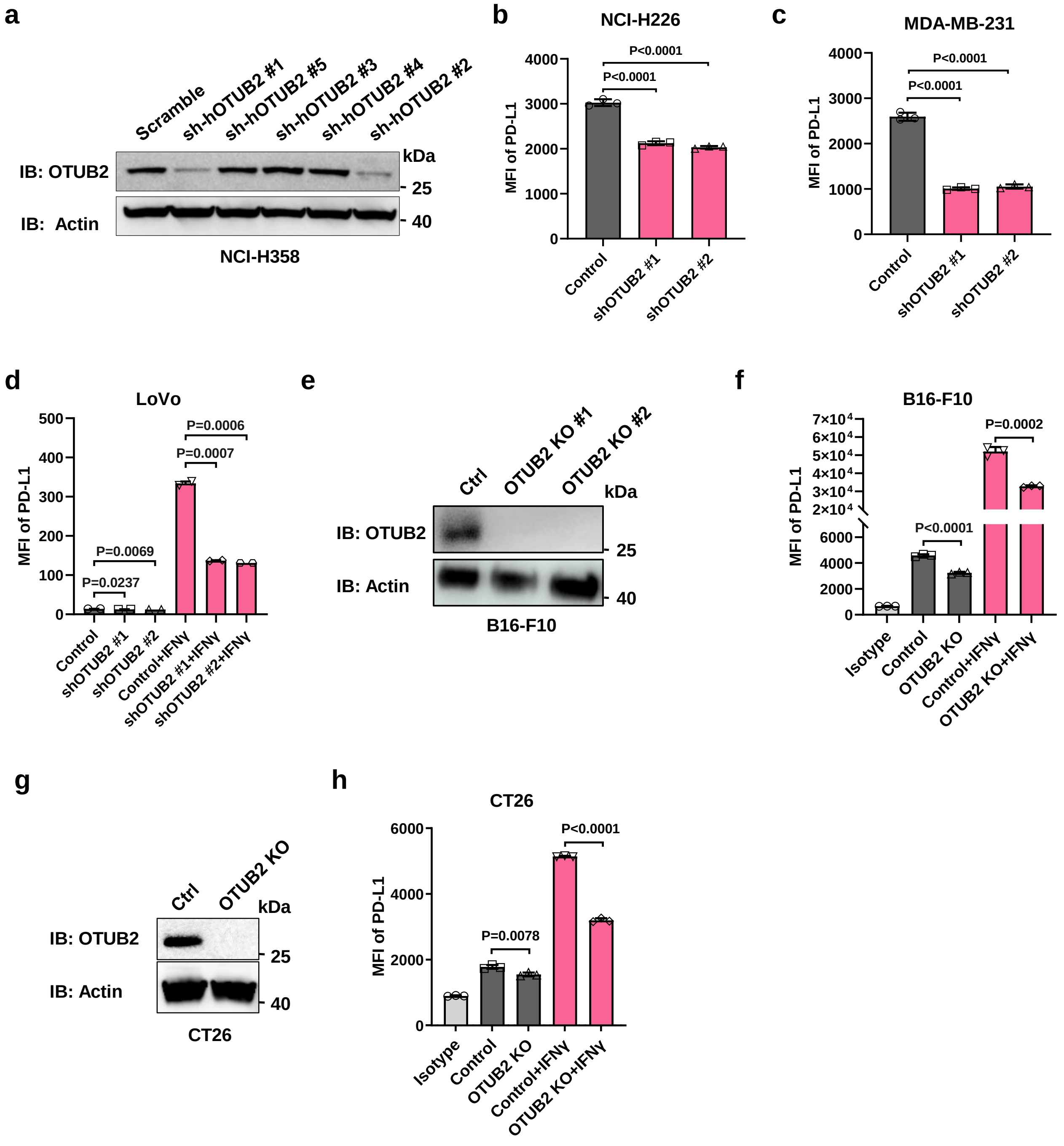

**Supplementary Fig. 8. Effects of OTUB2 KD or KO on the expression of PD-L1.** **a,** Knockdown (KD) efficiency of five shRNAs targeting human OTUB2. OTUB2 protein levels in NCI-H358 tumor cells expressing control shRNA (Scramble) or OTUB2-specific shRNAs (shRNA #1-#5). **b,** Flow cytometry analysis of PD-L1 levels on the surface of control and OTUB2-KD NCI-H226 cells. MFI, median fluorescence intensity. n = 3 biologically independent experiments. **c,** Flow cytometry analysis of PD-L1 levels on the surface of control and OTUB2-KD MDA-MB-231 cells. n = 3 biologically independent experiments. **d,** Flow cytometry analysis of PD-L1 levels on the surface of control and OTUB2-KD LoVo cells treated with or without IFN-γ. n = 2 biologically independent experiments. **e,** Western blot analysis of two independent OTUB2-KO B16-F10 clones (OTUB2 KO #1 and #2). **f,** Flow cytometry analysis of PD-L1 levels on the surface of control and OTUB2-KO B16-F10 cells treated with or without IFN-γ. n = 3 biologically independent experiments. **g,** Western blot analysis of the expression of OTUB2 in CT26 tumor cell lines with and without OTUB2 KO. **h,** Flow cytometry analysis of PD-L1 levels on the surface of control and OTUB2-KO CT26 cells treated with or without IFN-γ. n = 3 biologically independent experiments. The experiments were repeated three times independently with similar results. The data are presented as mean ± s.e.m., and P values were calculated using an unpaired two-sided Student's t test (**b**,**c**,**d**,**f**,**h**). Source data are provided as a Source Data file.

# Supplementary Fig. 9

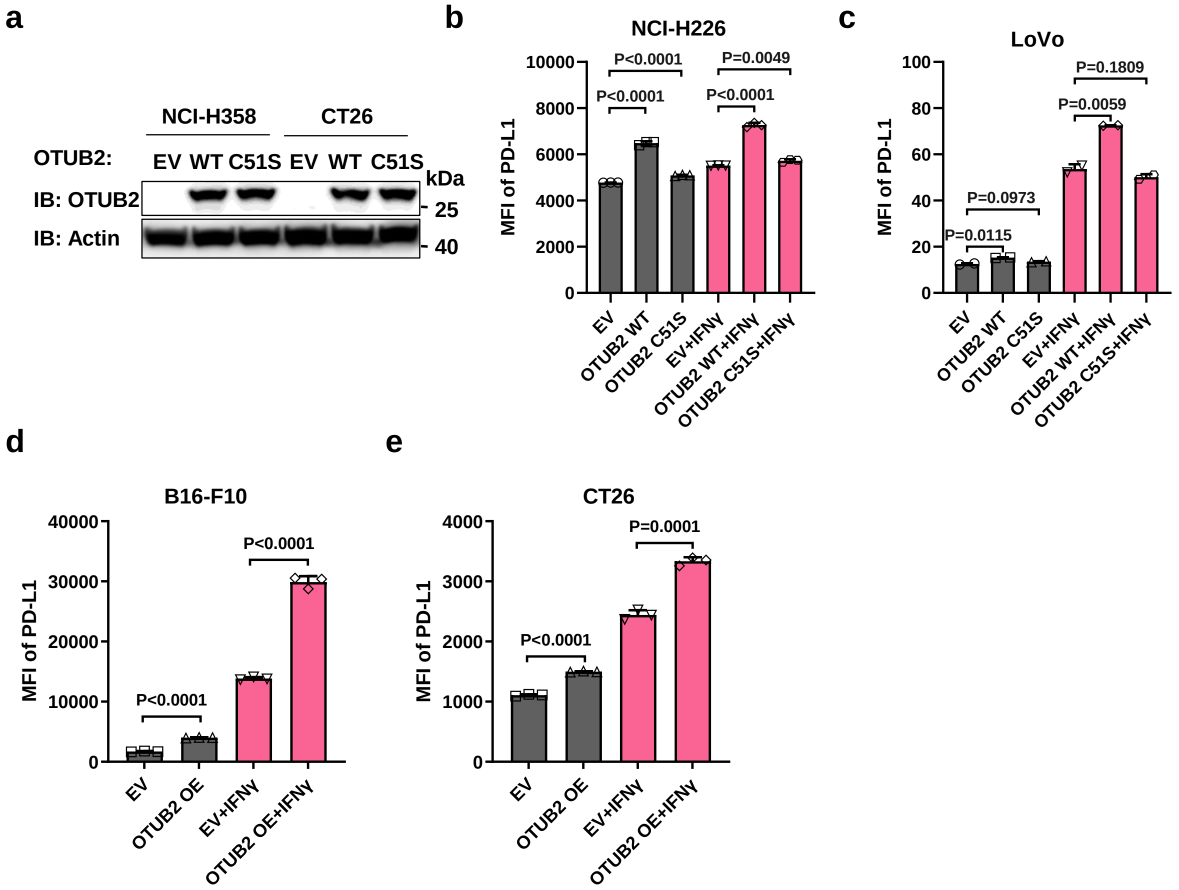

**Supplementary Fig. 9. Effects of OTUB2 OE on the expression of PD-L1. a,** Western blot analysis of the expression of OTUB2-WT and C51S in NCI-H358 and CT26 tumor cell lines. **b,** Flow cytometry analysis of PD-L1 levels on the surface of control and OTUB2-OE NCI-H226 cells treated with or without IFN-γ. MFI, median fluorescence intensity. n = 3 biologically independent experiments. **c,** Flow cytometry analysis of PD-L1 levels on the surface of control and OTUB2-OE LoVo cells treated with or without IFN-γ. n = 2 biologically independent experiments. **d,** Flow cytometry analysis of PD-L1 levels on the surface of control and OTUB2-OE B16-F10 cells treated with or without IFN-γ. n = 3 biologically independent experiments. **e,** Flow cytometry analysis of PD-L1 levels on the surface of control and OTUB2-OE CT26 cells treated with or without IFN-γ. n = 3 biologically independent experiments. The experiments were repeated three times independently with similar results. The data are presented as mean ± s.e.m., and P values were calculated using an unpaired two-sided Student's t test (**b-e**). Source data are provided as a Source Data file.

## Supplementary Fig. 10

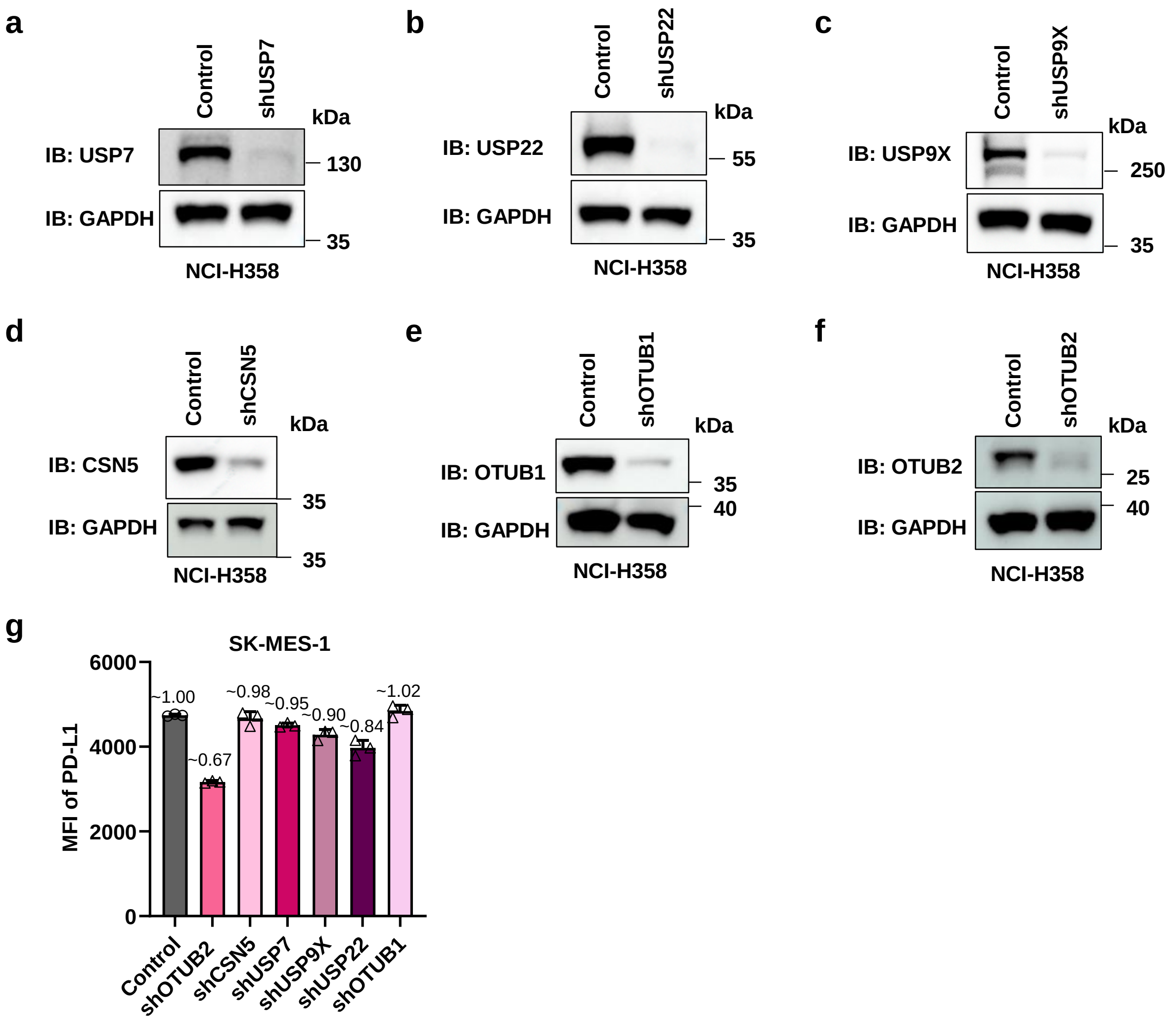

**Supplementary Fig. 10. Knockdown (KD) efficiency of shRNAs targeting different DUBs. a,** USP7 protein levels in NCI-H358 tumor cells expressing control shRNA or a USP7-specific shRNA. **b,** USP22 protein levels in NCI-H358 tumor cells expressing control shRNA or a USP22-specific shRNA. **c,** USP9X protein levels in NCI-H358 tumor cells expressing control shRNA or a USP9X-specific shRNA. **d,** CSN5 protein levels in NCI-H358 tumor cells expressing control shRNA or a CSN5-specific shRNA. **e,** OTUB1 protein levels in NCI-H358 tumor cells expressing control shRNA or an OTUB1-specific shRNA. **f,** OTUB2 protein levels in NCI-H358 tumor cells expressing control shRNA or an OTUB2-specific shRNA. **g,** Screening of other five DUBs that regulate PD-L1 protein level. SK-MES-1 cells individually were infected with shRNAs targeting different DUBs, and the surface expression of PD-L1 was detected. Each value above the bar is expressed as a fold change relative to the control. n = 3 biologically independent experiments. The experiments were repeated twice independently with similar results. Source data are provided as a Source Data file.

# Supplementary Fig. 11

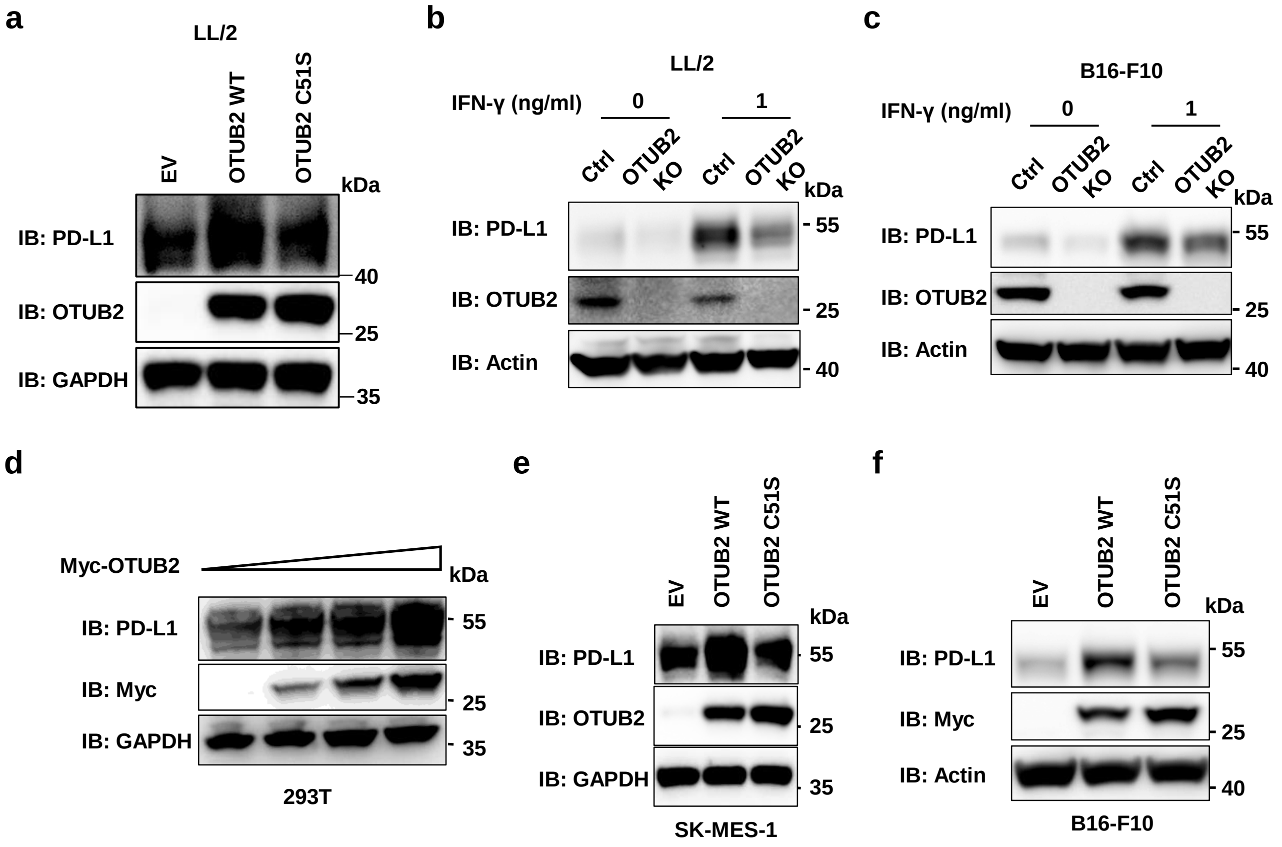

**Supplementary Fig. 11. Effects of OTUB2 OE or KO on the expression of PD-L1. a,** Western blot analysis of PD-L1 in LL/2 cells overexpressing a control vector (EV), OTUB2 (WT) or the OTUB2 C51S mutant (C51S). **b,** Western blot analysis of PD-L1 in control and OTUB2-KO LL/2 cells treated with or without IFN-γ. **c,** Western blot analysis of PD-L1 in control and OTUB2-KO B16-F10 cells treated with or without IFN-γ. **d,** Western blot analysis of endogenous PD-L1 in 293T cells transfected with increasing amounts of OTUB2. **e,** Western blot analysis of PD-L1 in SK-MES-1 cells overexpressing a control vector (EV), OTUB2 (WT) or the OTUB2 C51S mutant (C51S). **f,** Western blot analysis of PD-L1 in B16-F10 cells overexpressing a control vector (EV), OTUB2 (WT) or the OTUB2 C51S mutant (C51S). Source data are provided as a Source Data file.

## Supplementary Fig. 12

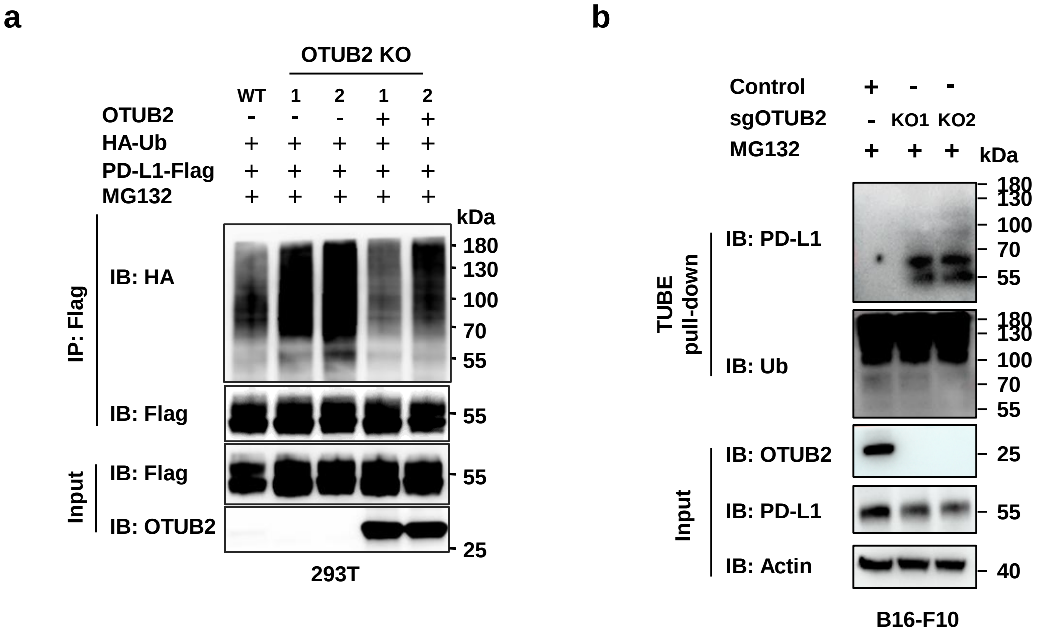

**Supplementary Fig. 12. Effects of OTUB2 on the polyubiquitination of PD-L1. a,** Determination of PD-L1 polyubiquitination levels in OTUB2-KO 293T cells after re-expression of OTUB2-WT. Immunoprecipitation was performed with an anti-Flag antibody**. b,** Determination of endogenous PD-L1 polyubiquitination levels in OTUB2-KO B16-F10 cells using a TUBE pull-down assay. Source data are provided as a Source Data file.

# Supplementary Fig. 13

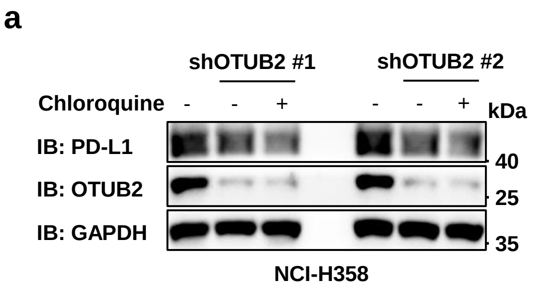


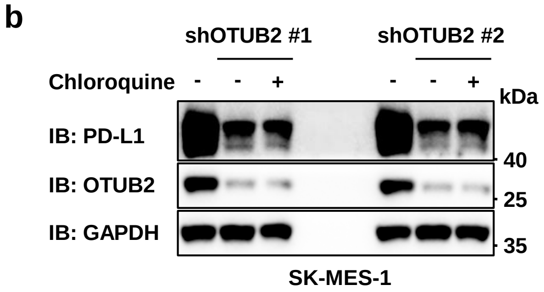

**Supplementary Fig. 13. Immunoblot analysis of PD-L1 in lung cancer cells treated with or without the lysosomal inhibitor chloroquine. a,** NCI-H358 tumor cells with or without OTUB2 KD in the absence or presence of 20 μM chloroquine (lysosome inhibitor) were collected to analyze PD-L1 expression. **b,** SK-MES-1 tumor cells with or without OTUB2 KD in the absence or presence of 20 μM chloroquine (lysosome inhibitor) were collected to analyze PD-L1 expression. Source data are provided as a Source Data file.

# Supplementary Fig. 14

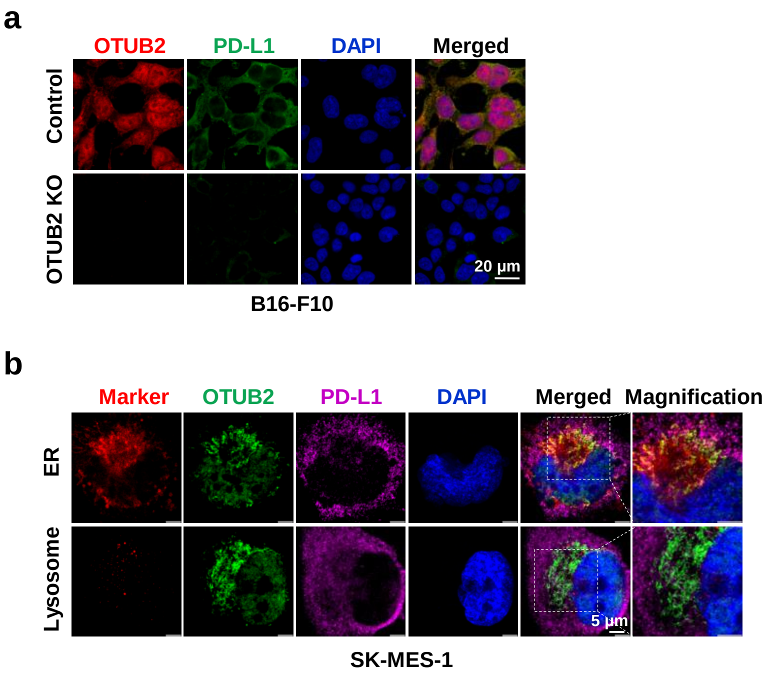

**Supplementary Fig. 14. The colocalization of PD-L1 and OTUB2. a,** B16-F10 tumor cells with or without OTUB2 KO were immunostained with an anti-OTUB2 antibody (red), an anti-PD-L1 antibody (green), and DAPI (blue). Scale bar, 20 µm. **b,** SK-MES-1 tumor cells were immunostained with an anti-PD-L1 antibody (purple), an anti-OTUB2 antibody (green), DAPI (blue) and lyso- or ER-specific trackers (red). Scale bar, 5 µm.

## Supplementary Fig. 15

a

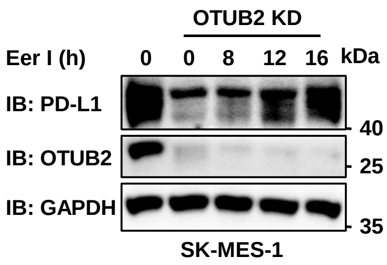


b

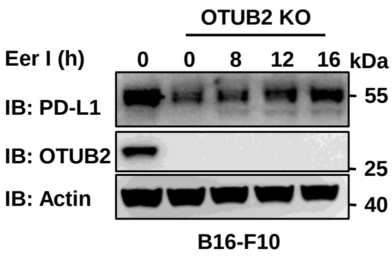


c

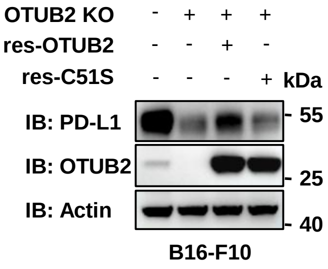

**Supplementary Fig. 15. OTUB2 regulates PD-L1 abundance through ERAD pathway. a,b,** SK-MES-1 cells (**a**) or B16-F10 tumor cells (**b**) with or without OTUB2 KD/KO in the absence or presence of 20 μM Eer I were collected to analyze PD-L1 expression at the indicated times. **c,** Determination of the PD-L1 levels in OTUB2-KO B16-F10 cells after re-expression of OTUB2-WT or OTUB2-C51S. Source data are provided as a Source Data file.

## Supplementary Fig. 16

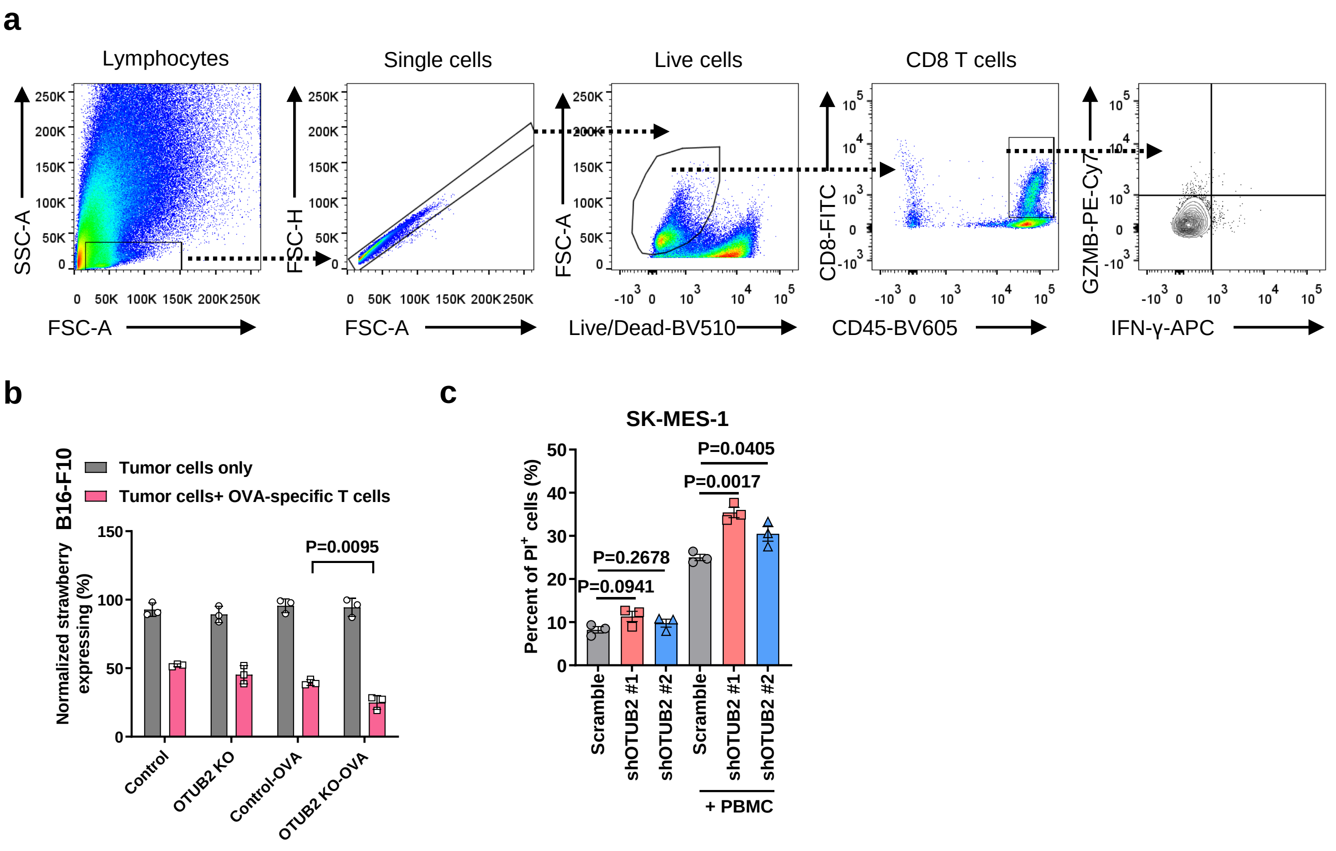

**Supplementary Fig. 16. Depletion of OTUB2 enhances intratumoral T-cell infiltration and tumor cell sensitivity to CD8$^+$ T-cell-mediated cytotoxicity. a,** Representative flow cytometry gating strategy for quantifying the numbers of various effector immune cell subsets in mouse tumors. **b,** Quantitative estimates of the fractions of live vector control and OTUB2-KO B16-F10-Strawberry tumor cells remaining after 24 h of incubation with activated OVA-specific T cells. **c,** Flow cytometry analysis of the peripheral blood mononuclear cell (PBMC)-mediated killing of vector control or OTUB2-KD SK-MES-1 tumor cells using propidium iodide (PI) staining. n = 3 biologically independent experiments. The above experiments were repeated three times independently with similar results. The data are presented as the mean ± s.e.m., and P values were calculated using an unpaired two-sided Student’s t test (**b** and **c**).

# Supplementary Fig. 17

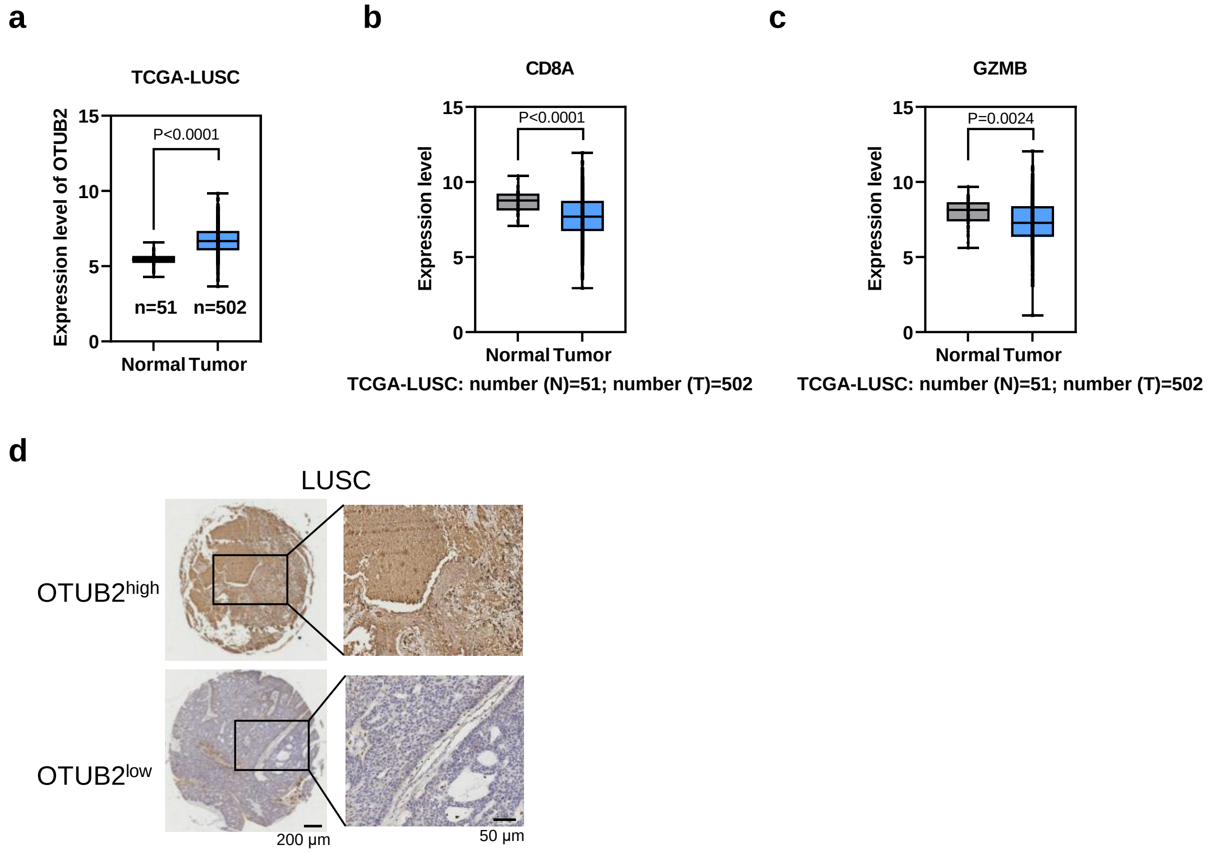

**Supplementary Fig. 17. The expression of OTUB2 is positively correlated with PD-L1 abundance. a,** Bioinformatic analyses of *OTUB2* mRNA expression in TCGA-LUSC patients (n = 502) relative to that in healthy controls (n = 51). **b,c,** Bioinformatic analyses of *CD8A* (**b**) and *GZMB* (**c**) mRNA expression in TCGA-LUSC patients (n = 502) relative to that in healthy controls (n = 51). **d**, Representative images of IHC staining of LUSC cancer tissues with high or low OTUB2 expression. Scale bars, 200 μm or 50 μm. The data are presented as the mean ± s.e.m., and P values were calculated using an unpaired two-sided Student's t test (**a**-**c**).

## Supplementary Fig. 18

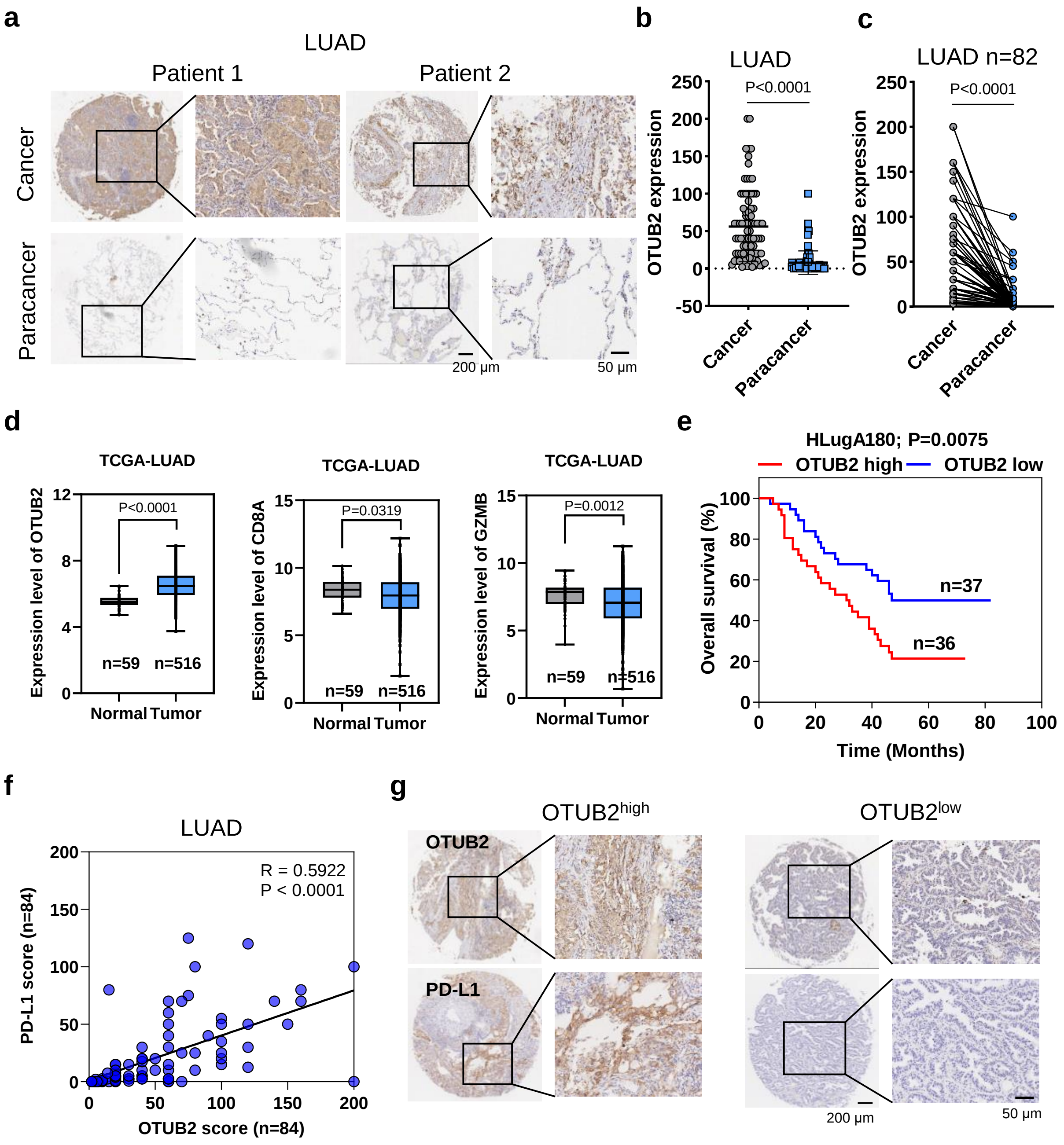

**Supplementary Fig. 18. The expression of OTUB2 is positively correlated with PD-L1 abundance in LUAD patient samples. a,** Representative images of IHC staining for OTUB2 in cancer tissues and paracancerous tissues from 86 LUAD patients. Scale bars, 200 µm or 50 µm. **b,** Comparison of OTUB2 expression between the cancer tissues (n=82) and paracancerous tissues (n = 82) of LUAD patients. **c,** OTUB2 expression in the paired tissue samples of 82 LUAD patients. **d,** Bioinformatic analyses of OTUB2, CD8A and GZMB expression in TCGA-LUAD patients (n = 516) relative to that in healthy controls (n = 59). **e**, Kaplan–Meier curve for the overall survival of LUAD patients based on OTUB2 expression scores calculated by IHC staining. Patients were divided into a high OTUB2 group (n = 36) or low OTUB1 group (n = 37). **f,** Pearson correlation analysis to determine the association between OTUB2 and PD-L1 by IHC staining scores (n = 84). **g,** Representative images of IHC staining for PD-L1 in LUAD cancer tissues with high or low OTUB2 expression. Scale bars, 200 µm or 50 µm. The data are presented as the mean ± s.e.m., and P values were calculated using an unpaired or paired two-sided Student's t test (**b**-**d**) except for in **e,** in which P values were calculated by the log-rank test, and **f,** in which P values were calculated by Spearman's correlation test.

## Supplementary Fig. 19

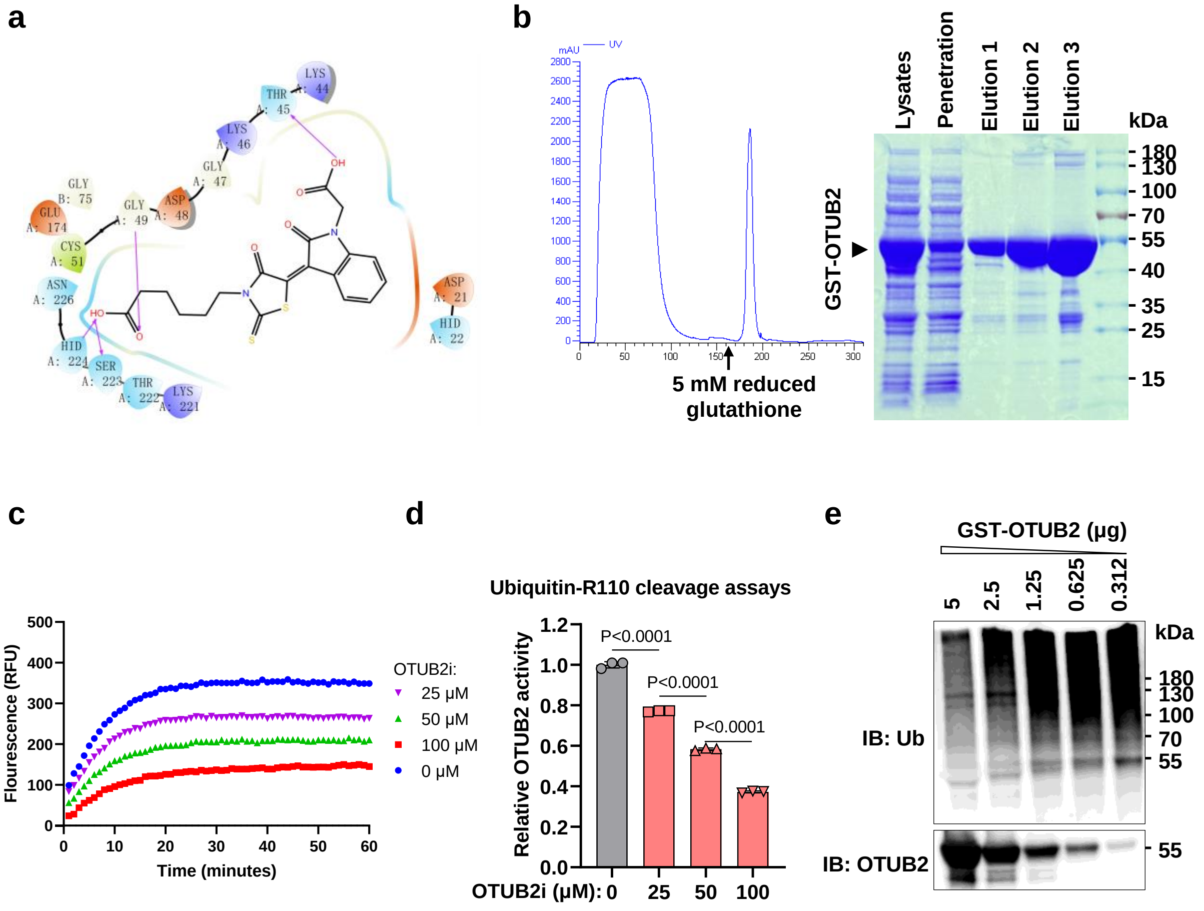

**Supplementary Fig. 19. Interaction between OTUB2 and OTUB2-IN-1 and characteristics of prokaryotically expressed GST-OTUB2 proteins. a,** A molecular docking model of the compound OTUB2-IN-1 and the catalytic pocket of OTUB2. **b,** Purification of GST-OTUB2 proteins using glutathione-based affinity purification and SDS–PAGE analysis of the purity of the GST-OTUB2 proteins. **c,** Deubiquitinating activity measured by ubiquitin-R110 cleavage assay. Ub-R110 was incubated with GST-OTUB2 in the absence or presence of varying concentrations of OTUB2-IN-1 (OTUB2i) (100, 50 or 25 μM). Deubiquitinating activity was determined by measuring R110 fluorescence as a function of time by fluorescence. **d,** Quantitative estimates of relative OTUB2 activity based on ubiquitin-R110 cleavage. n = 3 biologically independent experiments. **e,** Evaluation of the enzymatic activity of GST-OTUB2 proteins in deubiquitinating PD-L1 *in vitro*. The data are presented as the mean ± s.e.m., and P values were calculated using an unpaired two-sided Student's t test (**d**). Source data are provided as a Source Data file.

Supplementary Fig. 20

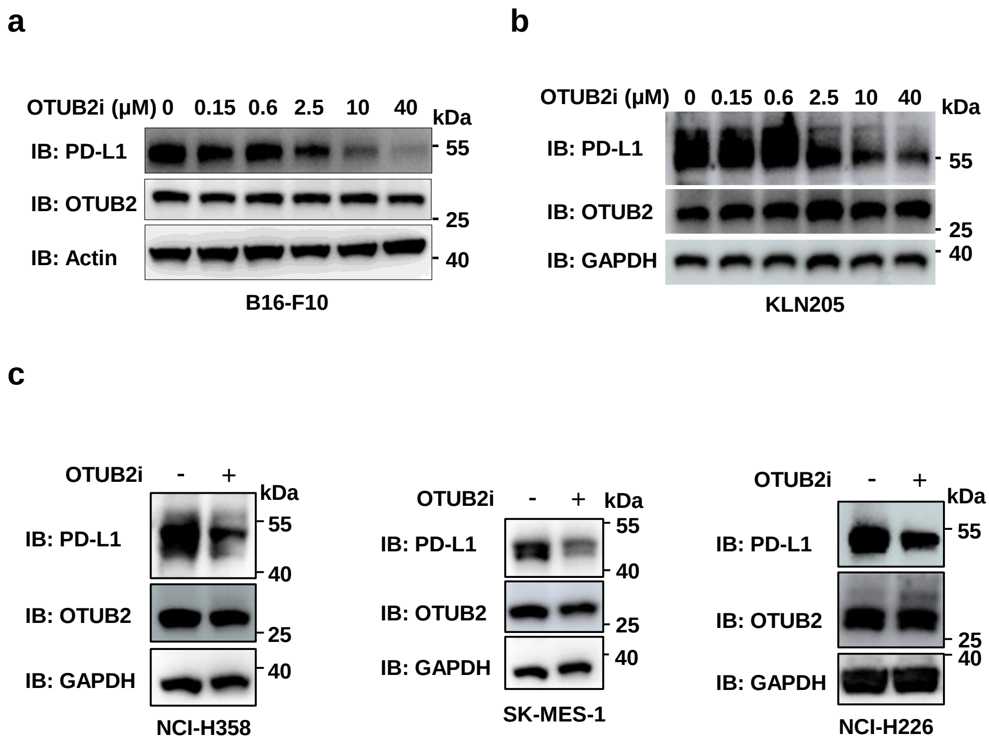

**Supplementary Fig. 20. Effects of OTUB2-IN-1 on PD-L1 stability in different tumor cells. a,** Inhibitory effects of OTUB2-IN-1 on the cellular level of PD-L1 in B16-F10 tumor cells. **b,** Inhibitory effects of OTUB2-IN-1 on the cellular level of PD-L1 in KLN205 tumor cells. **c,** Inhibitory effects of OTUB2-IN-1 (10 μM) on the cellular level of PD-L1 in multiple human lung cancer cell lines (NCI-H358, SK-MES-1 and NCI-H226). Source data are provided as a Source Data file.

## Supplementary Fig. 21

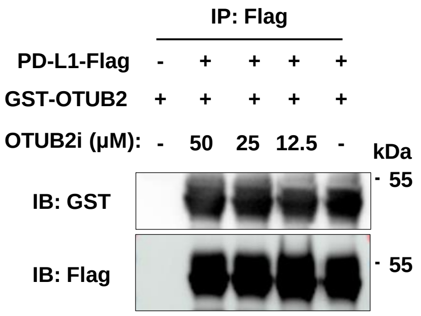

**Supplementary Fig. 21. Determine the ability of OTUB2-IN-1 to block the binding of OTUB2 to PD-L1.** The *in vitro* interaction between purified PD-L1-Flag proteins and GST-OTUB2 proteins in the presence of increasing concentration of the inhibitor examined by *in vitro* Flag pull-down assays. Source data are provided as a Source Data file.

## Supplementary Fig. 22

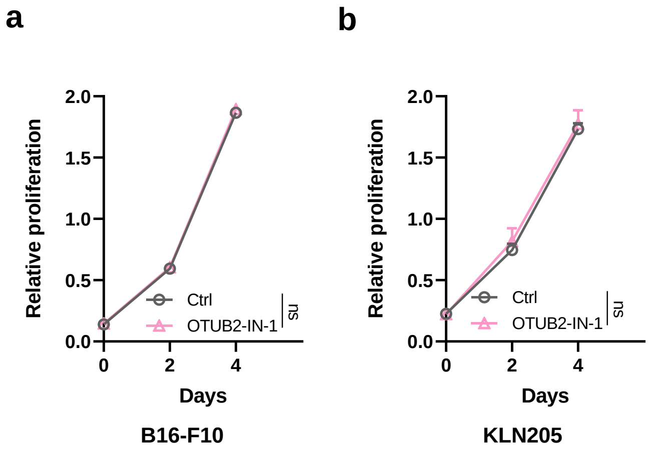

**Supplementary Fig. 22. Effects of OTUB2-IN-1 on tumor cell growth *in vitro*. a,** Effects of OTUB2-IN-1 (10 µM) on B16-F10 tumor cell growth. **b,** Effects of OTUB2-IN-1 (10 µM) on KLN205 tumor cell growth. n = 3 biologically independent experiments. The data are presented as the mean ± s.e.m., and P values were calculated using two-way analysis of variance (ANOVA). ns, not significant.

## Supplementary Fig. 23

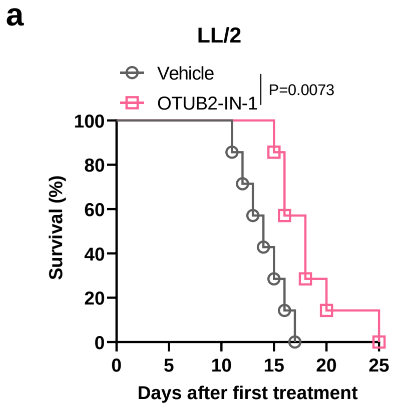


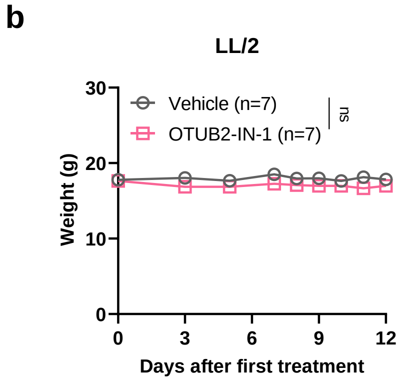

**Supplementary Fig. 23. Effects of OTUB2-IN-1 on LL/2 tumor growth. a,** Kaplan–Meier survival analysis of vehicle-or OTUB2-IN-1-treated host animals bearing LL/2 tumors. n = 7 mice per group. **b,** Weight monitoring of vehicle- or OTUB2-IN-1-treated host animals bearing LL/2 tumors. n = 7 mice per group. The data are presented as the mean ± s.e.m., and P values were calculated by the log-rank test (**a**), except for in **b,** in which P values were calculated using two-way analysis of variance (ANOVA). ns, not significant.

## Supplementary Fig. 24

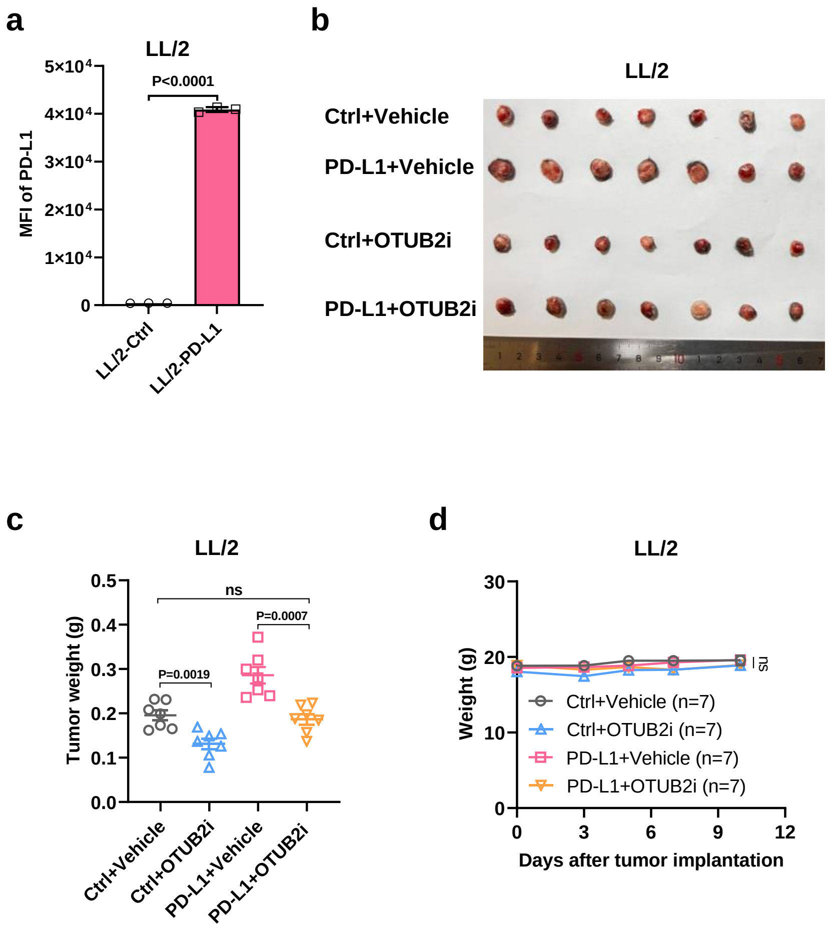

**Supplementary Fig. 24. Effects of OTUB2-IN-1 on PD-L1-overexpressing tumor cell growth *in vivo*. a,** Flow cytometry analysis of PD-L1 levels on the surface of control and PD-L1-overexpressing LL/2 tumor cells. n = 3 biologically independent experiments. **b,** Treatment with OTUB2-IN-1 reduced tumor growth in C57BL/6 mice implanted with control or PD-L1-overexpressing tumor cells. n = 7 mice per group. Photographs of tumors of each group at the end of the experiment. **c,** Tumor weight for each group at the end of the experiment. **d,** Weight monitoring of vehicle- or OTUB2-IN-1-treated host animals bearing control or PD-L1-overexpressing tumors. The data are presented as the mean ± s.e.m., and P values were calculated using an unpaired two-sided Student's t test (**a** and **c**), except for in **d,** in which P values were calculated using two-way analysis of variance (ANOVA). Source data are provided as a Source Data file. ns, not significant.

## Supplementary Fig. 25

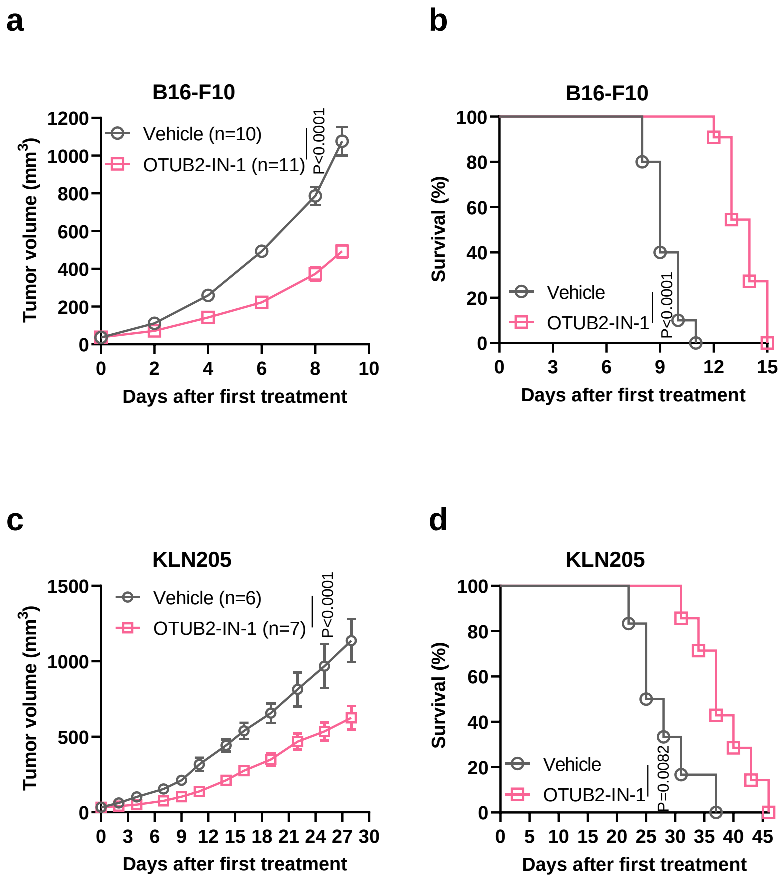

**Supplementary Fig. 25. Effects of OTUB2-IN-1 on tumor cell growth *in vivo*. a,** Treatment with OTUB2-IN-1 (20 mg/kg) reduced tumor growth in C57BL/6 mice implanted with B16-F10 tumor cells. n = 10 and 11 mice in the two groups. Mice were euthanized when the tumors reached 1000 $mm^3$ in volume or became necrotic. **b,** Kaplan–Meier survival analysis of vehicle- or OTUB2-IN-1-treated host animals bearing B16-F10 tumors. **c,** Treatment with OTUB2-IN-1 (20 mg/kg) reduced tumor growth in DBA/2 mice implanted with KLN205 tumor cells. n = 6 and 7 mice in the two groups. Mice were euthanized when the tumors reached 1000 $mm^3$ in volume or became necrotic. **d,** Kaplan–Meier survival analysis of vehicle- or OTUB2-IN-1-treated host animals bearing KLN205 tumors. The data are presented as the mean ± s.e.m., and P values were calculated using two-way analysis of variance (ANOVA) (**a** and **c**), except for in **b and d,** in which P values were calculated by the log-rank test.

## Supplementary Fig. 26

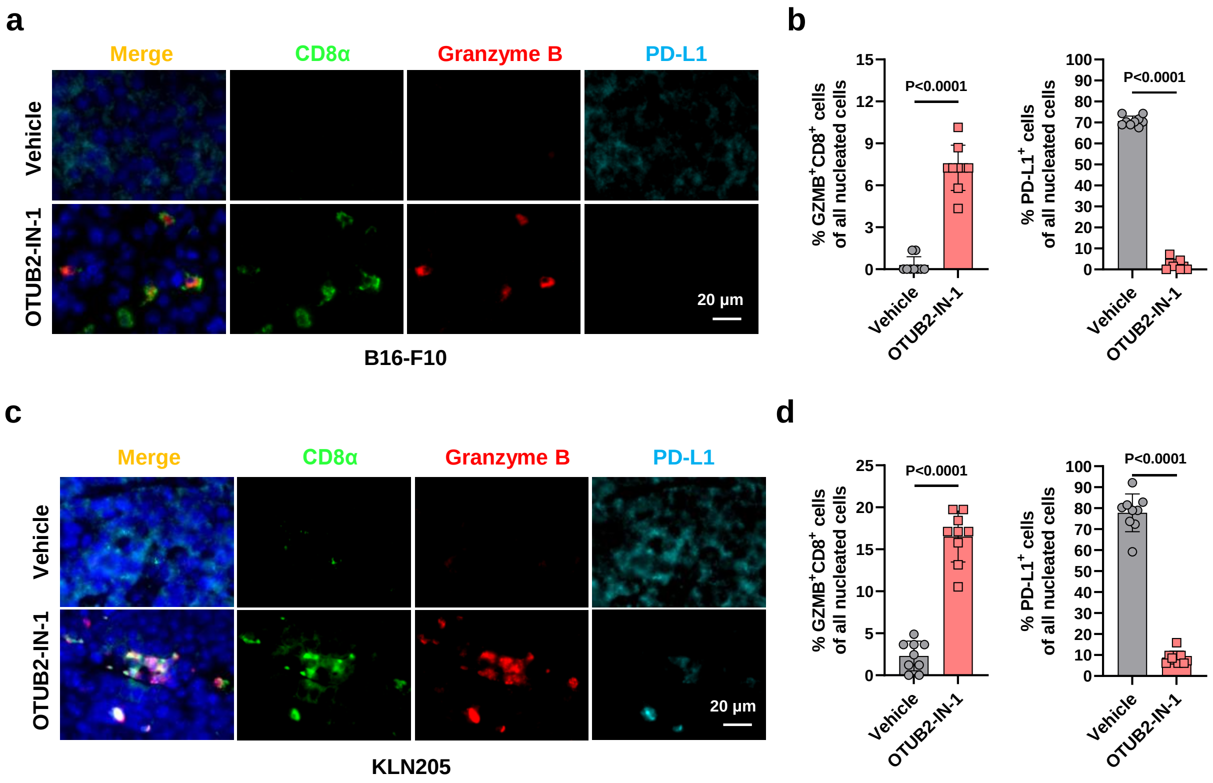

**Supplementary Fig. 26. Effects of OTUB2-IN-1 on regulating PD-L1 abundance and intratumoral infiltration of $CD8^+$ and $GZMB^+$ CTLs in mouse tumors. a,** Representative images of IHC staining with DAPI, anti-CD8α, anti-GZMB and anti-PD-L1 in B16-F10 tumors grown in syngeneic C57BL/6 mice after treatment with vehicle or OTUB2-IN-1. Scale bars, 20 μm. **b,** Quantitative estimates of $GZMB^+$ $CD8^+$ and PD-$L1^+$ cells in vehicle- or OTUB2-IN-1-treated tumors. Three fluorescent fields for each of the three samples were counted. n = 9 biologically independent samples. **c,** Representative images of IHC staining with DAPI, anti-CD8α, anti-GZMB and anti-PD-L1 in KLN205 tumors grown in syngeneic DBA/2 mice after treatment with vehicle or OTUB2-IN-1. Scale bars, 20 μm. **d,** Quantitative estimates of $GZMB^+$ $CD8^+$ and PD-$L1^+$ cells in vehicle- or OTUB2-IN-1-treated tumors. Three fluorescent fields for each of the three samples were counted. n = 9 biologically independent samples. The data are presented as the mean ± s.e.m., and P values were calculated by an unpaired two-sided Student's t test (**b** and **d**).

## Supplementary Fig. 27

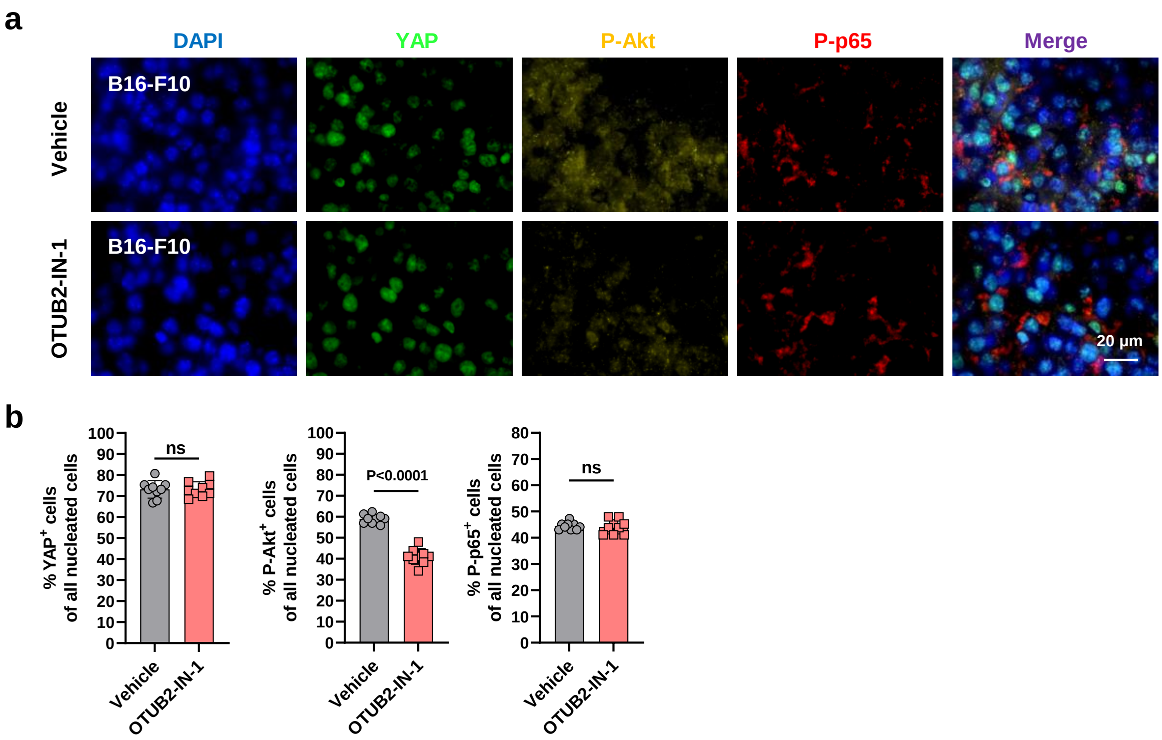

**Supplementary Fig. 27. Effects of OTUB2-IN-1 on regulating Hippo, NF-κB and Akt/mTOR pathways in B16-F10 tumors. a,** Representative images of IHC staining with DAPI, anti-YAP, anti-P-Akt and anti-P-p65 in B16-F10 tumors grown in syngeneic C57BL/6 mice after treatment with vehicle or OTUB2-IN-1. Scale bars, 20 μm. **b,** Quantitative estimates of $YAP^+$, $P\text{-}Akt^+$ and $P\text{-}p65^+$ cells in vehicle- or OTUB2-IN-1-treated tumors. Three fluorescent fields for each of the three samples were counted. n = 9 biologically independent samples. The data are presented as the mean ± s.e.m., and P values were calculated by an unpaired two-sided Student's t test (**b**). ns, not significant.

## Supplementary Fig. 28

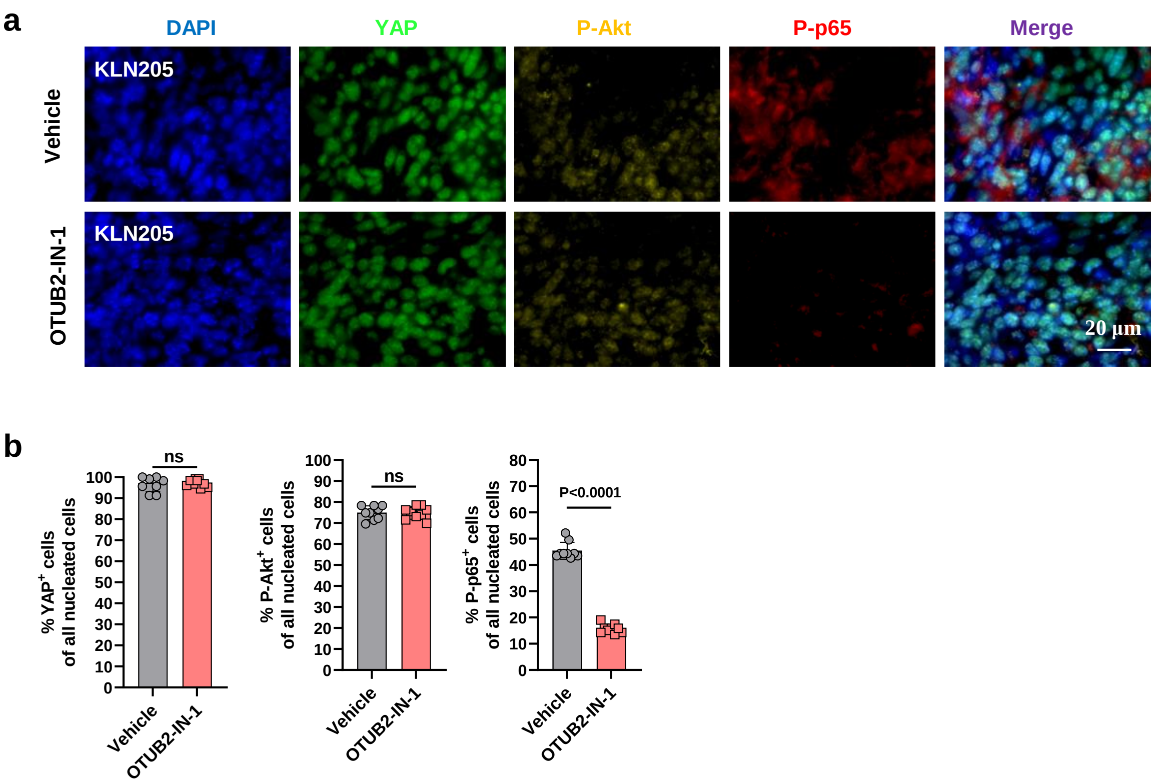

**Supplementary Fig. 28. Effects of OTUB2-IN-1 on regulating Hippo, NF-κB and Akt/mTOR pathways in KLN205 tumors. a,** Representative images of IHC staining with DAPI, anti-YAP, anti-P-Akt and anti-P-p65 in KLN205 tumors grown in syngeneic DBA/2 mice after treatment with vehicle or OTUB2-IN-1. Scale bars, 20 μm. **b,** Quantitative estimates of $YAP^+$, $P\text{-}Akt^+$ and $P\text{-}p65^+$ cells in vehicle- or OTUB2-IN-1-treated tumors. Three fluorescent fields for each of the three samples were counted. n = 9 biologically independent samples. The data are presented as the mean ± s.e.m., and P values were calculated by an unpaired two-sided Student's t test (**b**). ns, not significant.

## Supplementary Fig. 29

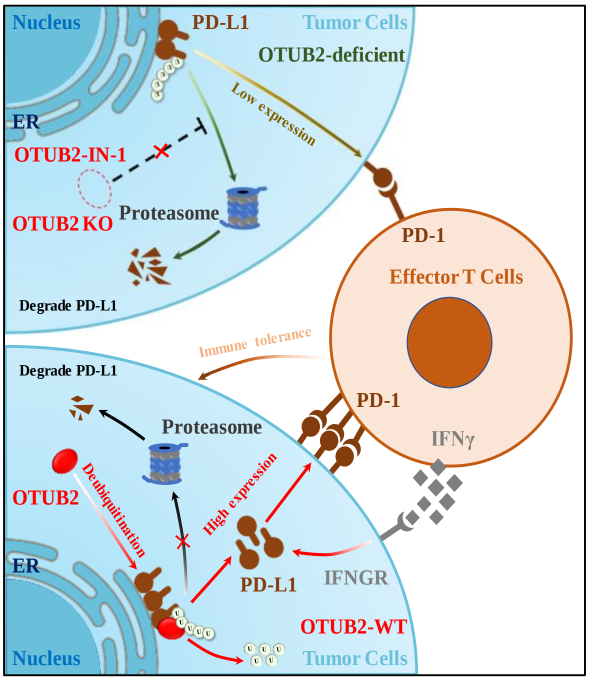

**Supplementary Fig. 29. Diagram illustrating OTUB2-mediated deubiquitination of PD-L1 in the ER.** Top, in the absence of OTUB2 (through genetic deletion or OUTB2 inhibitors), PD-L1 is transported into ERs and degraded. Down, in the presence of OTUB2, PD-L1 levels on the surface remain high and can thus reduce tumor cell sensitivity to $CD8^+$ T-cell-mediated cytotoxicity.

**Supplementary Table 1. DUBs gene list used in this study.**

| Ubiquitin-specific proteases (USPs) | Cysteine proteases ubiquitin C-terminal hydrolases (UCHs) | Ovarian tumor proteases (OTUs) | Machado-Joseph domain proteases (MJDs) | Metalloproteases JAB1/MPN/MOV34 (JAMM) | MCPIP family | Novel |
|---|---|---|---|---|---|---|
| USP1 | UCHL1 | ALG13 | ATXN3 | BRCC3 | KHNYN | PPPDE1 |
| USP2 | UCHL3 | OTUB1 | JOSD1 | COPS5 | N4BP1 | HAUSP |
| USP3 | UCHL5 | OTUB2 | JOSD2 | COPS6/CSN6 | NYNRIN | |
| USP4 | BAP1 | OTUD1 | ATXN3L | EIF3F | ZC3H12A | |
| USP5 | | OTUD3 | | EIF3H | ZC3H12B | |
| USP6 | | OTUD4 | | MPND | ZC3H12C | |
| USP6NL | | OTUD5 | | MYSM1 | ZC3H12D | |
| USP7 | | OTUD6A | | PRPF8 | | |
| USP8 | | OTUD6B | | PSMD14 | | |
| USP9X | | OTUD7A | | PSMD7 | | |
| USP9Y | | OTUD7B | | STAMBP | | |
| USP10 | | OTULIN | | STAMBPL1 | | |
| USP11 | | VCPIP1 | | | | |
| USP12 | | ZRANB1 | | | | |
| USP13 | | YOD1 | | | | |
| USP14 | | TNFAIP3 | | | | |
| USP15 | | | | | | |
| USP16 | | | | | | |
| USP17 | | | | | | |
| USP17L2 | | | | | | |
| USP17L6P | | | | | | |
| USP18 | | | | | | |
| USP19 | | | | | | |
| USP20 | | | | | | |
| USP21 | | | | | | |
| USP22 | | | | | | |
| USP24 | | | | | | |
| USP25 | | | | | | |
| USP26 | | | | | | |
| USP27X | | | | | | |
| USP28 | | | | | | |
| USP29 | | | | | | |
| USP30 | | | | | | |
| USP31 | | | | | | |
| USP32 | | | | | | |
| USP33 | | | | | | |
| USP34 | | | | | | |
| USP35 | | | | | | |
| USP36 | | | | | | |
| USP37 | | | | | | |
| USP38 | | | | | | |
| USP39 | | | | | | |
| USP40 | | | | | | |
| USP42 | | | | | | |
| USP43 | | | | | | |
| USP44 | | | | | | |
| USP45 | | | | | | |
| USP46 | | | | | | |
| USP47 | | | | | | |
| USP48 | | | | | | |
| USP49 | | | | | | |
| USP50 | | | | | | |
| USP51 | | | | | | |
| USP52 | | | | | | |
| USP53 | | | | | | |
| USP54 | | | | | | |
| CYLD | | | | | | |
| USPL1 | | | | | | |

**Supplementary Table 2. The docking scores of the compounds capable of binding to the catalytic pocket of OTUB2.**

| Item | Drug_Names | docking score | MW |
|---|---|---|---|
| 1 | (5-(2-amino-5-isobutylthiazol-4-yl)furan-2-yl)phosphonic acid | -6.945 | 302.29 |
| 2 | (E)-4-(4-(2-carboxyvinyl)phenylsulfonamido)butanoic acid | -6.811 | 313.33 |
| 3 | 2-((2-(3,4-dihydroxyphenyl)-2-oxoethyl)thio)nicotinic acid | -6.059 | 305.31 |
| 4 | (E)-2-(5-(2-carboxyvinyl)-2-methylphenylsulfonamido)benzoic acid | -5.864 | 361.37 |
| 5 | 3-(2-hydroxy-6-methoxyquinolin-3-yl)propanoic acid | -5.812 | 247.25 |
| 6 | 2,2'-((9H-fluorene-2,7-disulfonyl)bis(azanediyl))diacetic acid | -5.783 | 440.45 |
| 7 | (R)-2-(isobutylamino)-4-oxo-4-((4-sulfamoylphenyl)amino)butanoic acid | -5.691 | 343.40 |
| 8 | (2R,3R)-3-(1H-benzo[d]imidazol-2-yl)-2,3-dihydroxypropanoic acid | -5.656 | 222.20 |
| 9 | (R)-4-((3-(3-allylthioureido)phenyl)amino)-2-(butylamino)-4-oxobutanoic acid | -5.649 | 378.49 |
| 10 | (R)-4-((3-(3-allylthioureido)phenyl)amino)-2-(isobutylamino)-4-oxobutanoic acid | -5.613 | 378.49 |
| 11 | (R)-3-((3-(carboxymethyl)-2,4-dioxothiazolidin-5-yl)amino)benzoic acid | -5.582 | 310.28 |
| 12 | 2-(5-oxo-2,5-dihydro-1H-pyrazol-3-yl)acetic acid | -5.503 | 142.11 |
| 13 | 3-(4-amino-3-((2-((4-nitrophenyl)amino)-2-oxoethyl)thio)-5-oxo-4,5-dihydro-1,2,4-triazin-6-yl)propanoic acid | -5.363 | 394.36 |
| 14 | (E)-6-(5-(1-(carboxymethyl)-2-oxoindolin-3-ylidene)-4-oxo-2-thioxothiazolidin-3-yl)hexanoic acid | -5.345 | 434.49 |
| 15 | 2-(2-(3,5-dimethoxybenzamido)thiazol-4-yl)acetic acid | -5.286 | 322.34 |
| 16 | (R)-4-((3-(3-allylthioureido)phenyl)amino)-4-oxo-2-(pentylamino)butanoic acid | -5.281 | 392.52 |
| 17 | (R)-2-hydroxy-2-(pyridin-2-yl)propanoic acid | -5.279 | 167.16 |
| 18 | (Z)-2-(5-(4-hydroxy-3-methoxy-5-nitrobenzylidene)-4-oxo-2-thioxothiazolidin-3-yl)acetic acid | -5.259 | 370.36 |
| 19 | (R)-4-((3-(3-allylthioureido)phenyl)amino)-2-((furan-2-ylmethyl)amino)-4-oxobutanoic acid | -5.242 | 402.47 |
| 20 | 2-(2-((pyridin-4-ylmethyl)thio)thiazol-4-yl)acetic acid | -5.242 | 266.34 |

**Supplementary Table 3. shRNA sequences.**

| Gene | Sequence (5’-3’) |
|---|---|
| shControl | CCTAAGGTTAAGTCGCCCTCG |
| sh-mOTUB2 #1 | GATGAGGAGATGGACATCAAA |
| sh-mOTUB2 #2 | CTTCGGTTTATCTGCTCTATA |
| sh-mOTUB2 #3 | GAAGACCAAAGGAGACGGAAA |
| sh-mOTUB2 #4 | CTATCCATTCTTCGGGAT CAT |
| sh-hOTUB2 #1 | CCTATGTGTCACTGGATTATT |
| sh-hOTUB2 #2 | TGTGGTGGAACTGGTAGAGAA |
| sh-hOTUB2 #3 | CGAGATGGATACCGCCCTGAA |
| sh-hOTUB2 #4 | CATCCCACTACAACATCCTTT |
| sh-hOTUB2 #5 | TGTGGTGGAACTGGTAGAGAA |
| sh-hOTUB1 | AGCGACTCCGAAGGTGTTAAC |
| sh-hUSP22 | TGTGCCAGGACTACATCTATG |
| sh-hCSN5 | GAGCTGTTGTGGAATAAAT |
| sh-hUSP7 | CTCAGAACCCTGTGATCAA |
| sh-hUSP9X | CGCCTGATTCTTCCAATGAAA |

**Supplementary Table 4. sgRNA sequences.**

| Gene | Sequence (5’-3’) |
|---|---|
| sgControl | GAACGACTAGTTAGGCGTGTA |
| sg-mOTUB2 #1 | GCAAAAGATTCACCTCGATC |
| sg-mOTUB2 #1 | GGTAGAGTACGTCGACGAGA |
| sg-hOTUB2 #1 | TTCAACGACCAGAGTGCCT |

**Supplementary Table 5. Plasmids.**

| Plasmid | Source | Catalog No. |
|---|---|---|
| pLKO.1 | Addgene | 10878 |
| psPAX2 | Addgene | 12260 |
| PMD2.G | Addgene | 12259 |
| pCI-neo-cOVA | Addgene | 25097 |
| lentiCRISPR v2 | Addgene | 52961 |
| lentiCas9-Blast | Addgene | 52962 |

**Supplementary Table 6. Antibodies.**

| Antibody | Source | Catalog No. | Application (Dilution) |
|---|---|---|---|
| APC anti-mouse CD274 antibody | Biolegend | 124312 | FCM(1:200) |
| APC anti-human CD274 antibody | Biolegend | 329708 | FCM(1:200) |
| BV605 anti-mouse CD45 antibody | Biolegend | 103140 | FCM(1:200) |
| FITC mouse CD8a antibody | Biolegend | 100706 | FCM(1:200) |
| APC anti-mouse IFN-γ antibody | Biolegend | 505810 | FCM(1:200) |
| PE/Cyanine7 anti-human/mouse granzyme B recombinant antibody | Biolegend | 372214 | FCM(1:200) |
| Zombie Aqua Fixable Viability Kit | Biolegend | 423102 | FCM(1:200) |
| Anti-OTUB2 mouse monoclonal antibody | Sangon Biotech | D199590-0100 | WB(1:500) IP(1:100) |
| Anti-PD-L1-mouse | Abcam | ab213480 | WB(1:500) IP(1:100) |
| PD-L1 monoclonal antibody | Proteintech | 66248-1-Ig | WB(1:500) |
| Monoclonal anti-HA antibody | SIGMA-ALDRICH | H3663-200UL | WB(1:2,000) |
| Monoclonal anti-FLAG® M2 antibody | SIGMA-ALDRICH | F1804-200UG | WB(1:2000) |
| Anti-c-Myc antibody | SIGMA-ALDRICH | C3956 | WB(1:500) |
| Ubiquitin (P4D1) mouse mAb | CST | 3936 | WB(1:1,000) |
| beta actin antibody | Proteintech | 66009-1-Ig | WB(1:2,000) |
| GAPDH antibody | Proteintech | 60004-1-Ig | WB(1:2,000) |
| Goat Anti-Rabbit IgG (H + L)-HRP Conjugate | Bio-Rad | 1706515 | WB(1:3,000) |
| Goat Anti-Mouse IgG (H + L)-HRP Conjugate | Bio-Rad | 1706516 | WB(1:3,000) |
| anti-GST-HRP | Proteintech | HRP-66001 | WB(1:500) |
| anti-GST antibody | Sino Biological | 11213-RP01 | ELISA(1:2,000) |
| OTUB2 antibody | Affinity | AF9147 | IHC(1:3,000) |
| Calnexin (C5C9) rabbit mAb | CST | 2679S | IF(1:1,000) |
| Alexa Fluor® 488 donkey anti-mouse IgG (H+L) | Invitrogen | A-21202 | IF(1:2,000) |
| TRITC-conjugated goat anti-rabbit IgG | SIGMA-ALDRICH | T6778-1ML | IF(1:500) |
| Alexa Fluor 488-conjugated donkey anti-rabbit IgG | Invitrogen | A-21206 | IF(1:2000) |
| Alexa Fluor 647-conjugated goat anti-mouse IgG | Invitrogen | A-21237 | IF(1:500) |
| CD8α (D4W2Z) XP® rabbit mAb | CST | 98941 | IHC(1:200) |

| | | | |
|---|---|---|---|
| Anti-granzyme B antibody | Abcam | ab4059 | IHC(1:100) |
| Recombinant anti-CD8α antibody | Abcam | ab209775 | IHC(1:1000) |
| PD-L1 Polyclonal antibody | Proteintech | 17952-1-AP | IHC(1:1000) |
| OTUB1 | Abcam | ab175200 | WB(1:200) |
| CSN5 | CST | 6895S | WB(1:200) |
| USP9X | Proteintech | 55054-1-AP | WB(1:2000) |
| USP7 | CST | 4833T | WB(1:2000) |
| USP22 | Abcam | ab195289 | WB(1:200) |
| YAP | Affinity | DF3182 | IHC(1:100) |
| Phospho-AKT1 (S473) | Abcam | ab81283 | IHC(1:100) |
| Phospho-p65 (S536) | Affinity | AF2006 | IHC(1:100) |
| InVivoMAb anti-mouse CD8α | Bio X Cell | BE0061 | 200 μg/mouse |
| InVivoMAb rat IgG2b LTF-2 | Bio X Cell | BE0090 | 200 μg/mouse |

**Supplementary Table 7. Reagents and chemical compounds.**

| Reagents | Source | Catalog No. |
|---|---|---|
| Recombinant Murine IFN-γ | BEYOTIME | P6137-100μg |
| Recombinant Human IFN-gamma Protein | R&D Systems | 285-IF-100 |
| Fixation/Permeabilization Solution Kit | BD | 554714 |
| Pierce™ IP Lysis Buffer | Thermo Scientific | 87787 |
| Dynabeads® Protein A | Invitrogen | 100-02D |
| ANTI-FLAG® M2 Affinity Gel | SIGMA-ALDRICH | A2220 |
| Pierce anti-HA Magnetic Beads | Thermo Scientific | 88836 |
| Lumi-Light$^{PLUS}$ Western Blotting Substrate | Roche | 12015196001 |
| Glutathione sepharose 4B | GE | 17075601 |
| Protease Inhibitor Cocktail | MCE | HY-K0011 |
| DAPI | Invitrogen | D1306 |
| Cycloheximide | MCE | HY-12320 |
| MG-132 | MCE | HY-13259 |
| Chloroquine diphosphate | MCE | HY-17589 |
| Eeyarestatin I | MCE | HY-110078 |
| SIINFEKL peptide | Sangon Biotech | |
| mouse recombinant IL-2 | Sino Biological | 51061-MNAE-20 |
| Dynabeads™ Human T-Activator CD3/CD28 | GIBCO | 11131D |
| Human Recombinant IL-2 | stemcell | 78036 |
| ER-Tracker™ Red | Thermo Fisher | E34250 |
| Lyso-Tracker Red | BEYOTIME | C1046 |
| OTUB2-IN-1 | Life Chemicals | F0444-0064 |
| OPAL 6-PLEX manual detection kit | AKOYA | NEL811001KT |
| Rhodamine 110-Ub | R&D Systems | U-550-050 |
| 1,10-phenanthroline | MCE | HY-W004544 |
| N-Ethylmaleimide | Topscience | T3088 |
| PR-619 | MCE | HY-13814 |
| TUBE2 magnetic beads | LifeSensors | UM-0402M-1000 |

## Uncropped blots for Supplementary Fig. 6a

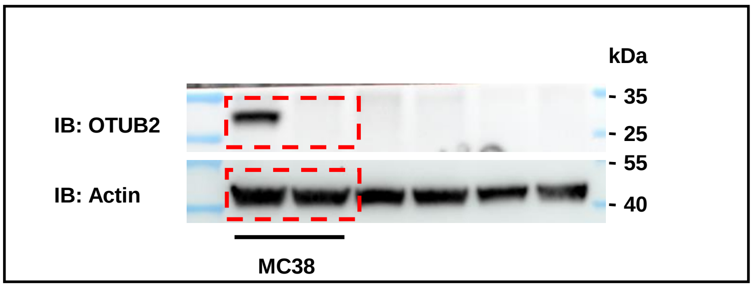


## Uncropped blots for Supplementary Fig. 6b

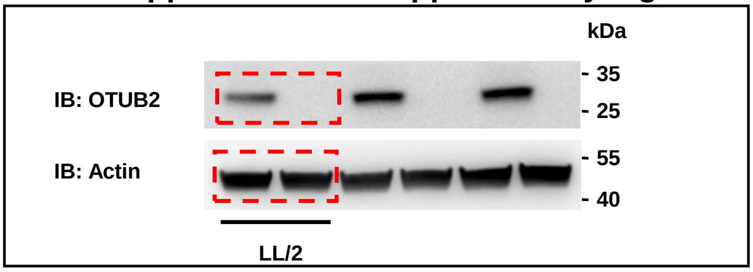


## Uncropped blots for Supplementary Fig. 6c

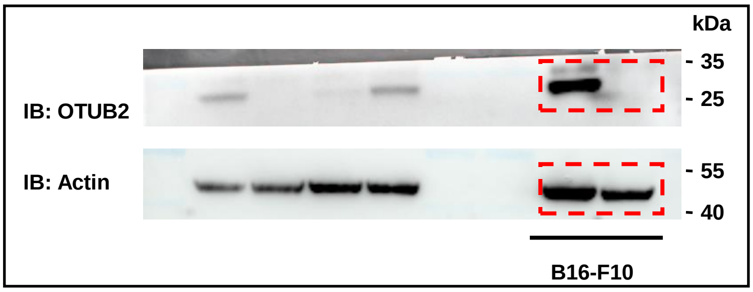


## Uncropped blots for Supplementary Fig. 6g

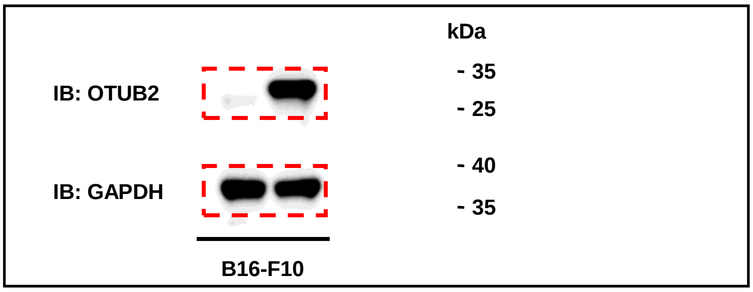

## Uncropped blots for Supplementary Fig. 7a

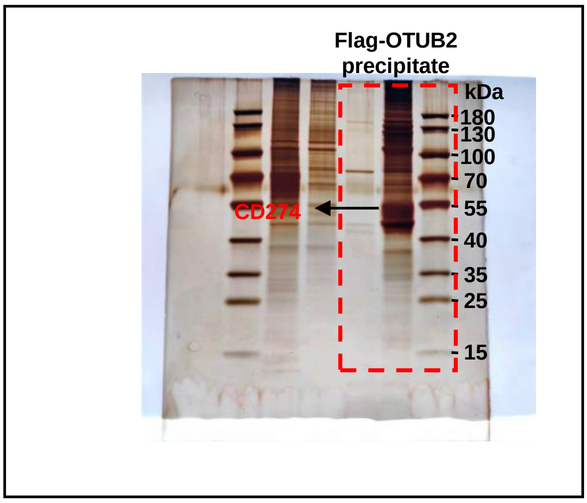


## Uncropped blots for Supplementary Fig. 7b

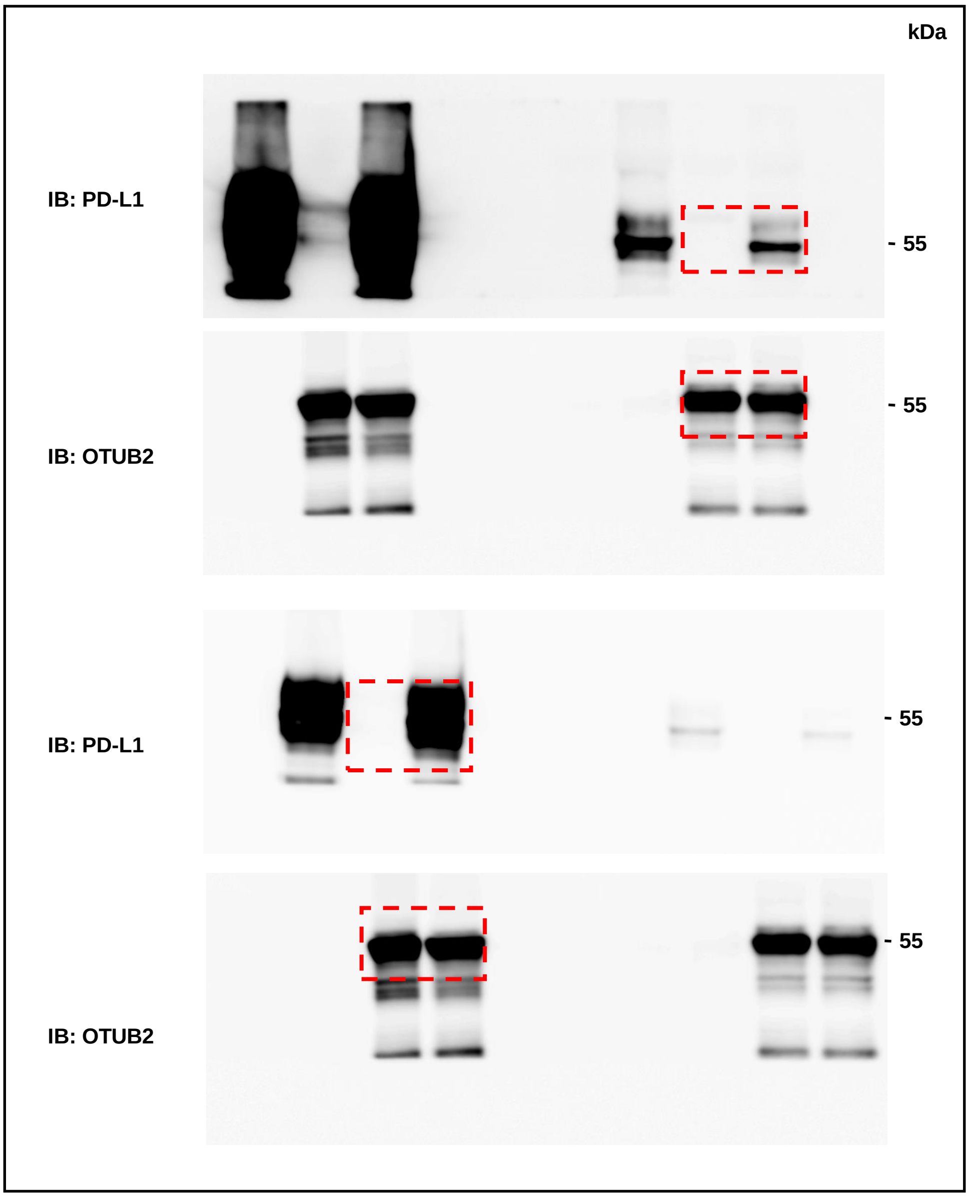

## Uncropped blots for Supplementary Fig. 7c

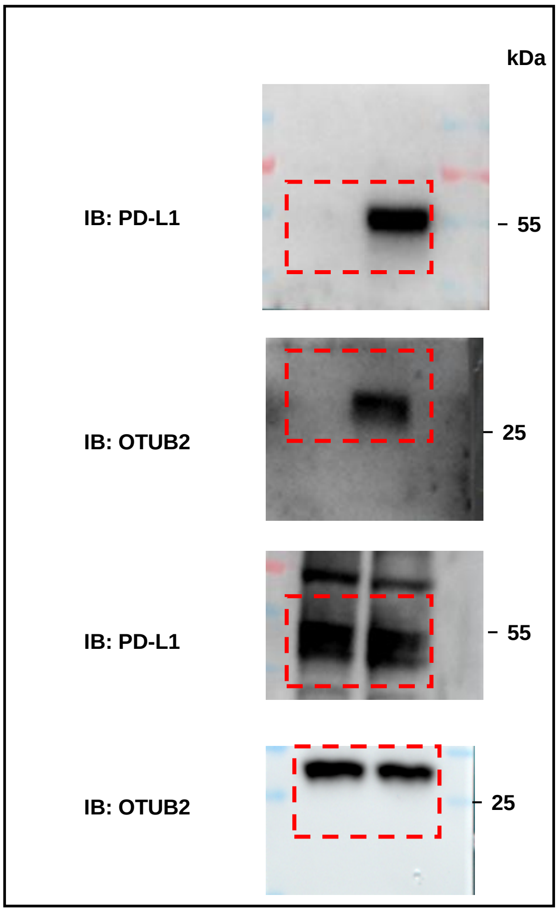


## Uncropped blots for Supplementary Fig. 7e

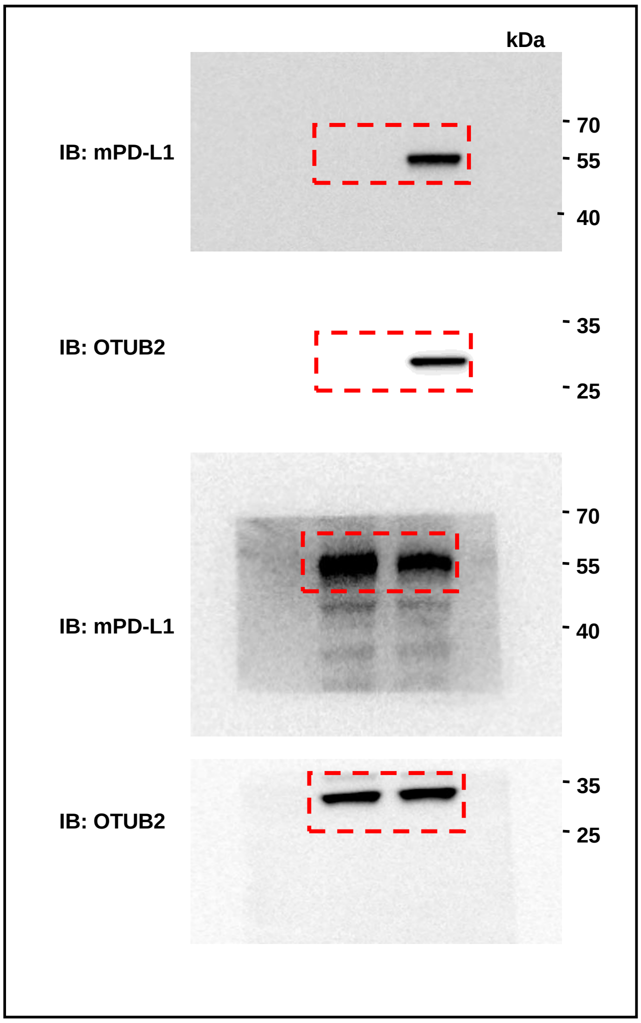


## Uncropped blots for Supplementary Fig. 7d

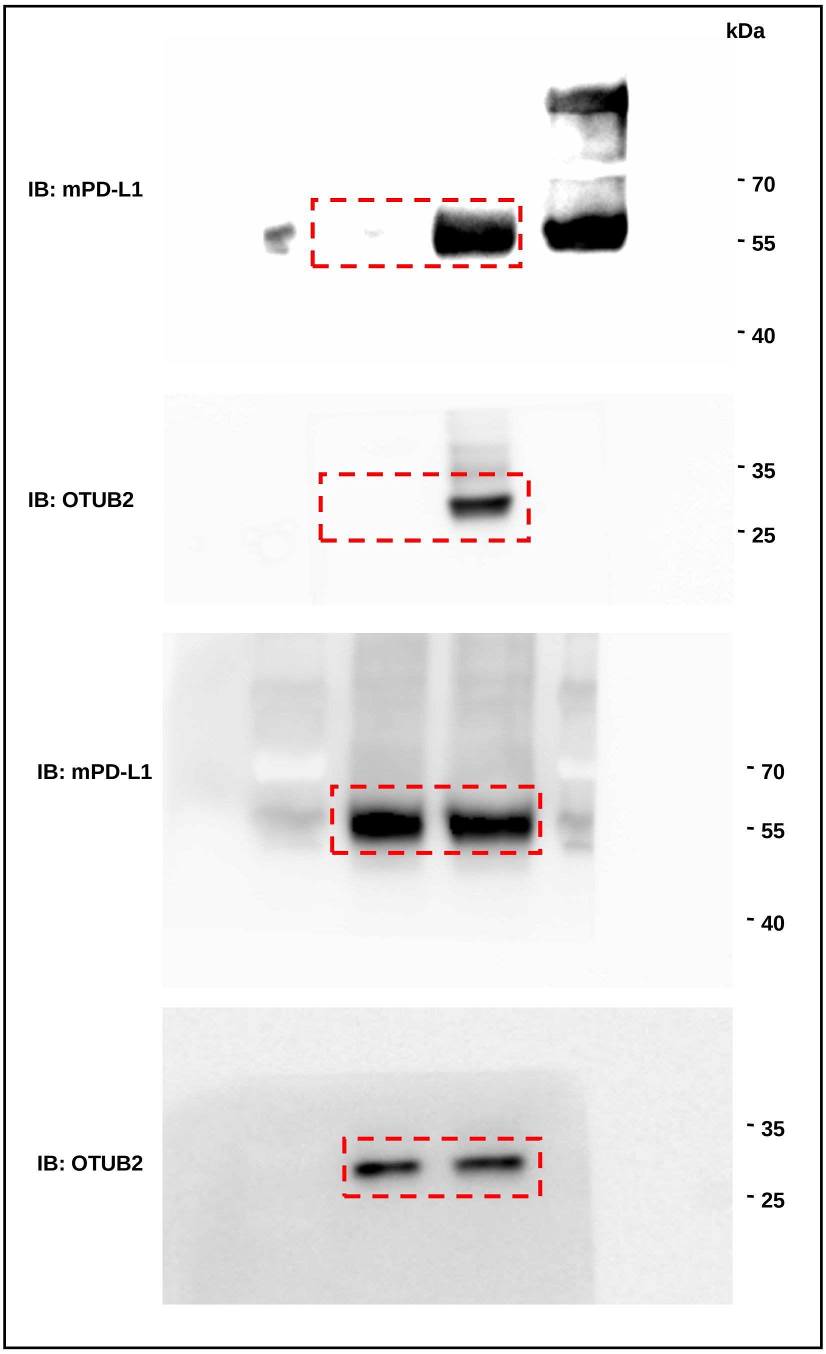

## Uncropped blots for Supplementary Fig. 8a

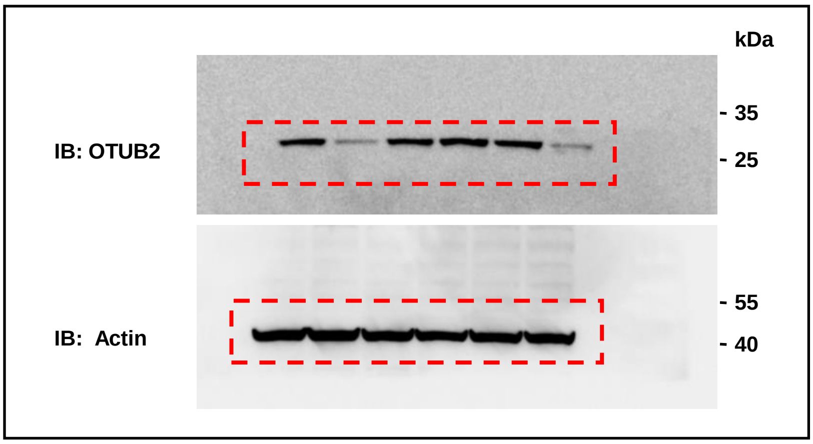


## Uncropped blots for Supplementary Fig. 8e

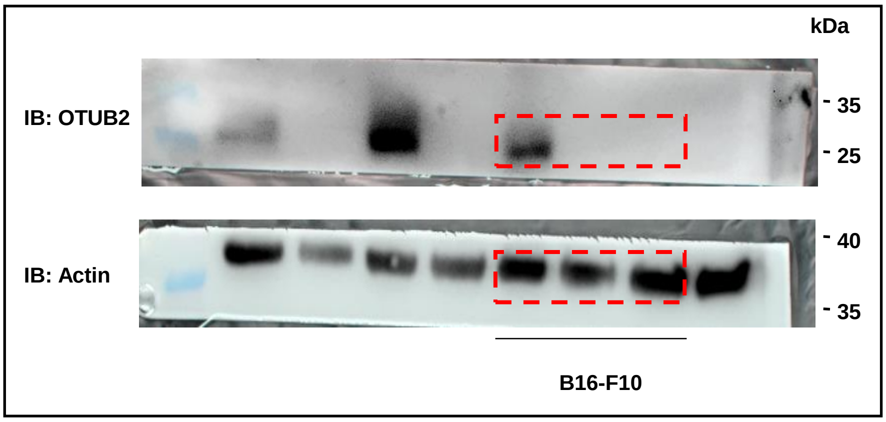


## Uncropped blots for Supplementary Fig. 8g

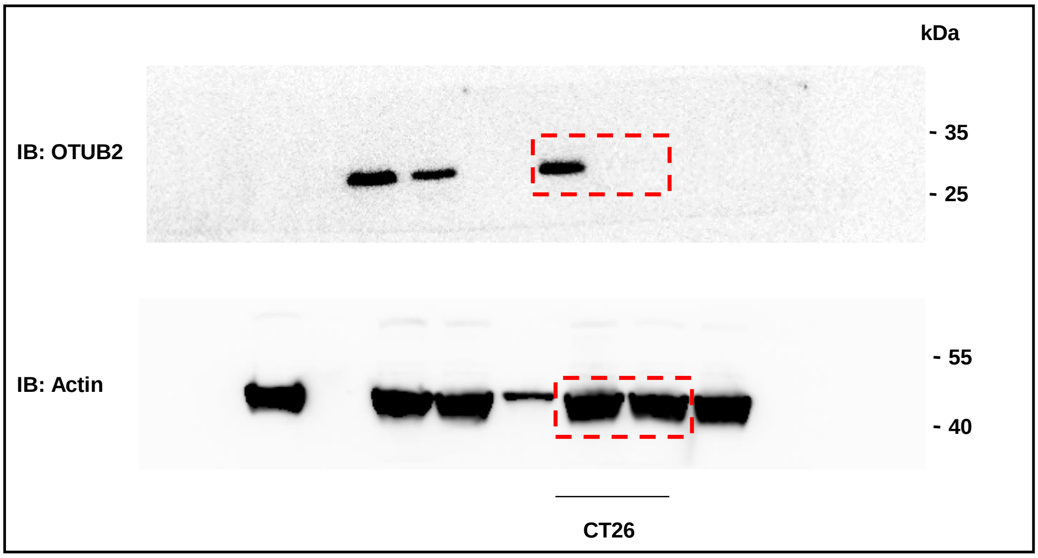

## Uncropped blots for Supplementary Fig. 9a

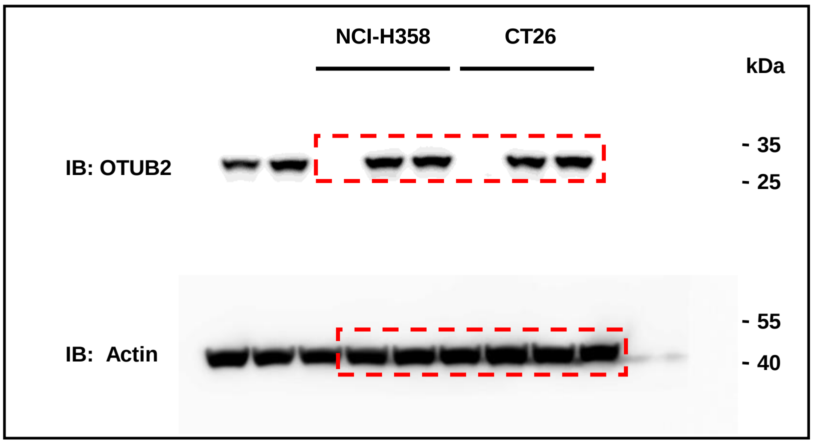

## Uncropped blots for Supplementary Fig. 10a

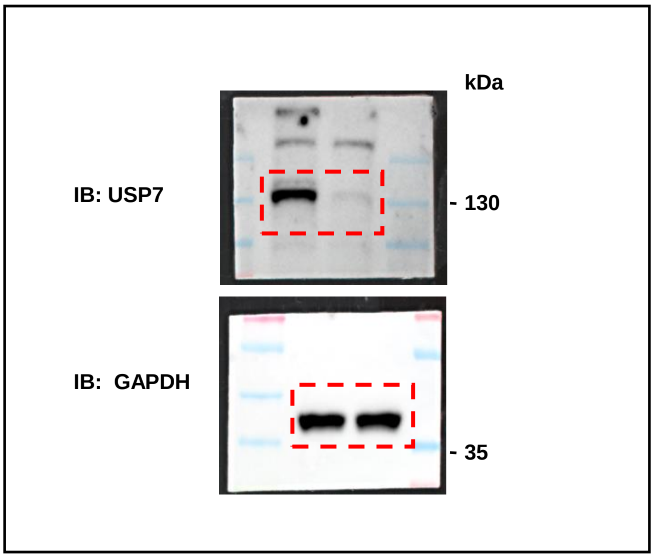


## Uncropped blots for Supplementary Fig. 10b

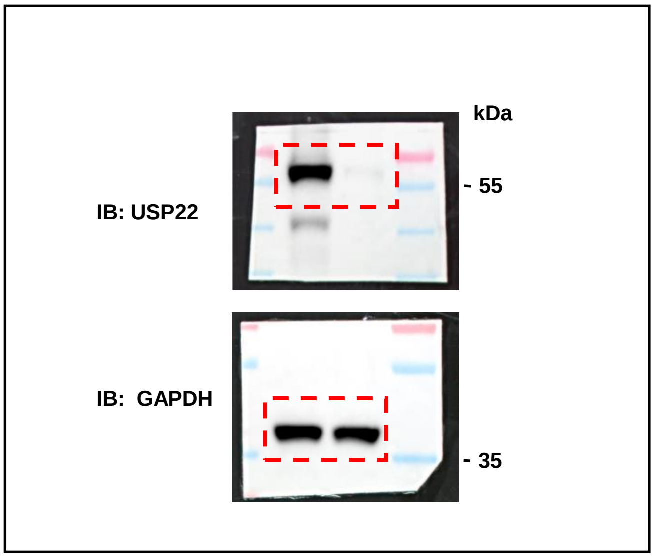


## Uncropped blots for Supplementary Fig. 10c

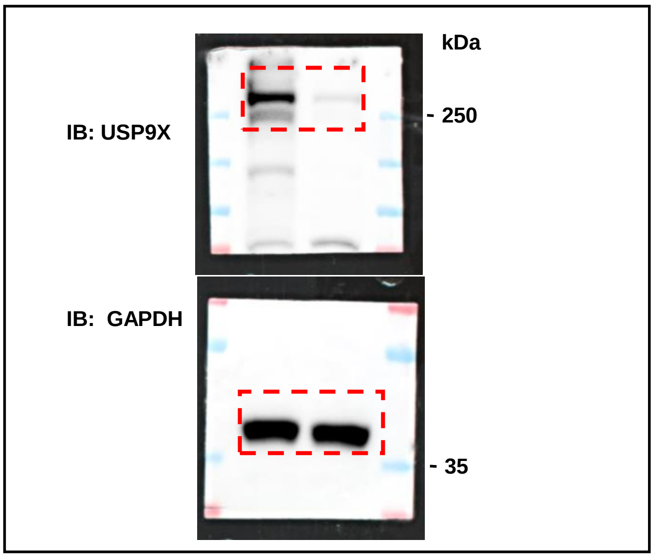


## Uncropped blots for Supplementary Fig. 10d

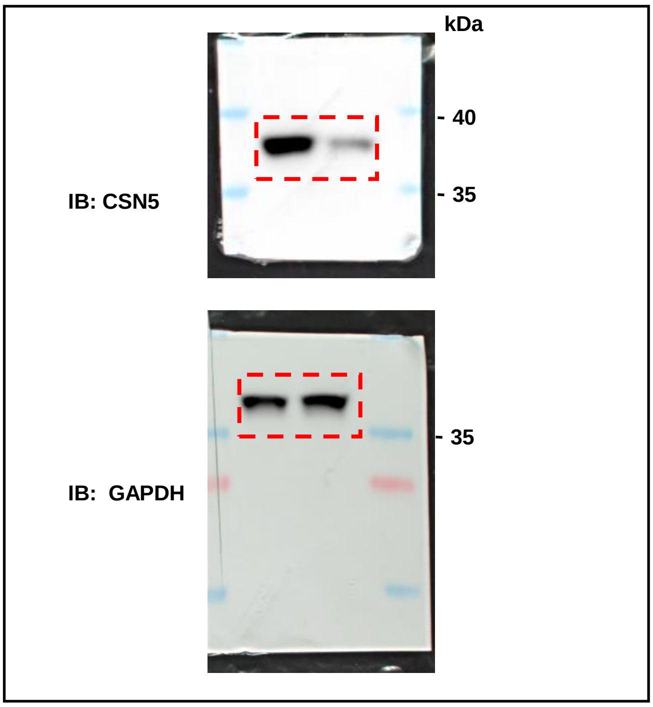


## Uncropped blots for Supplementary Fig. 10e

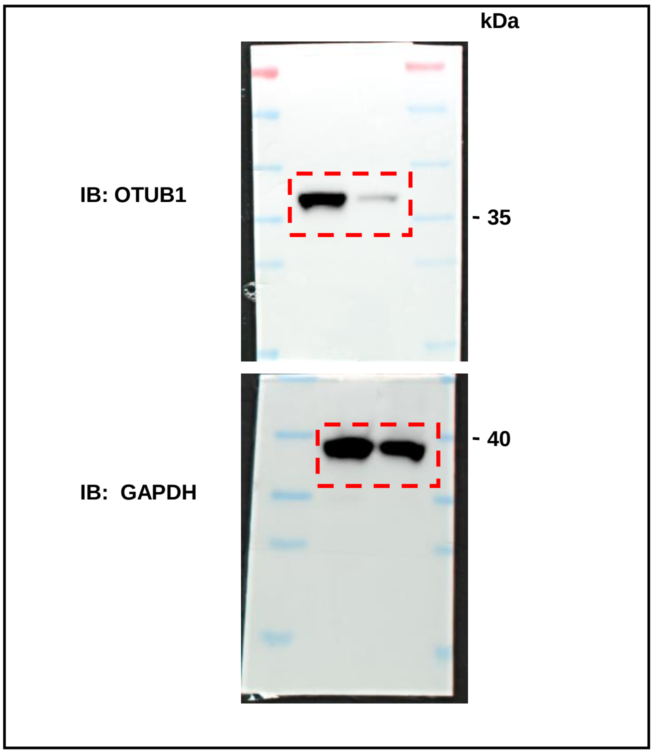


## Uncropped blots for Supplementary Fig. 10f

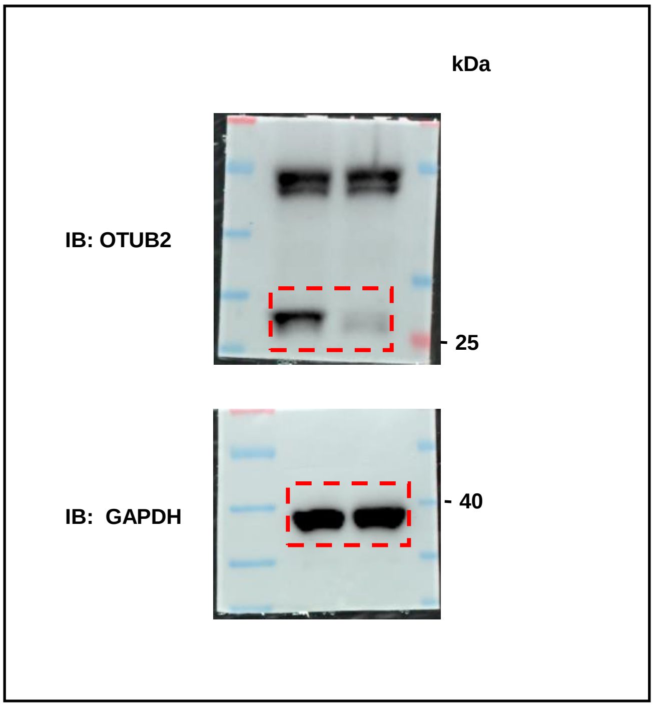

## Uncropped blots for Supplementary Fig. 11a

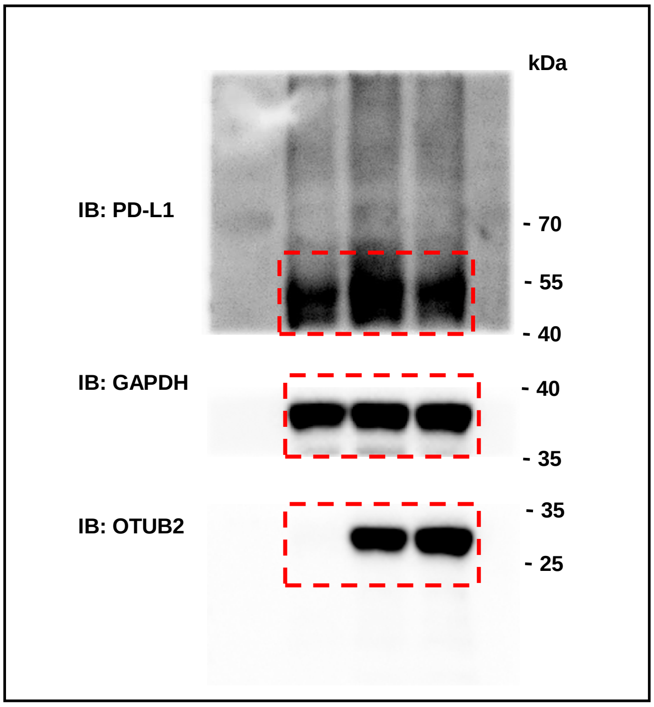


## Uncropped blots for Supplementary Fig. 11b

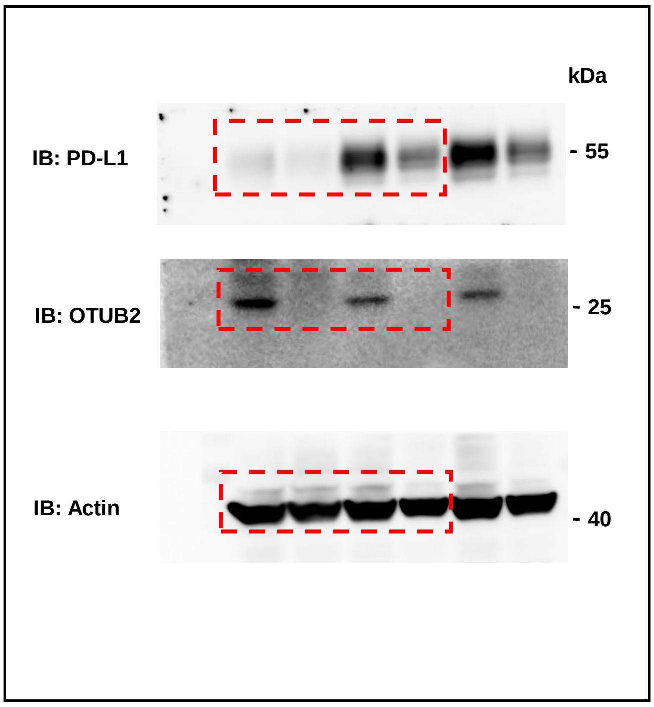


## Uncropped blots for Supplementary Fig. 11c

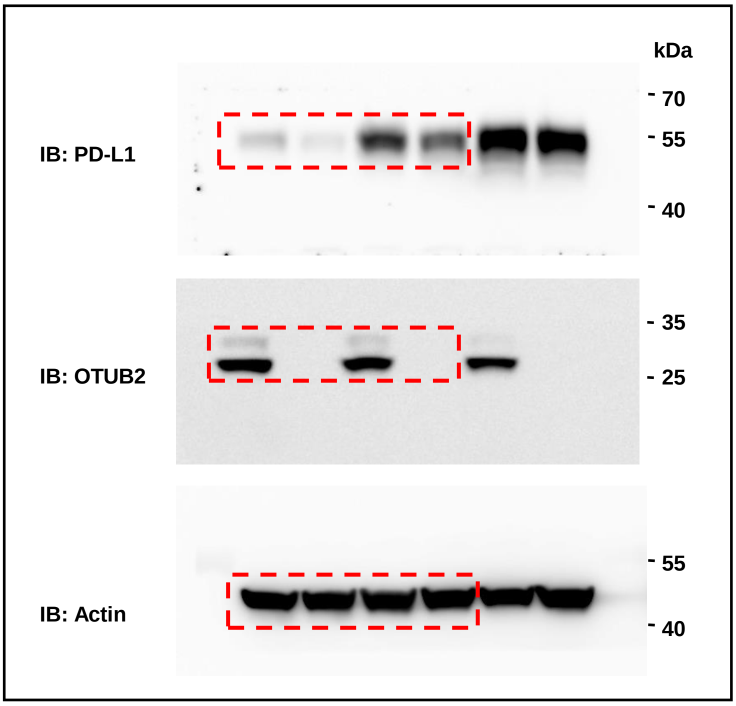


## Uncropped blots for Supplementary Fig. 11d

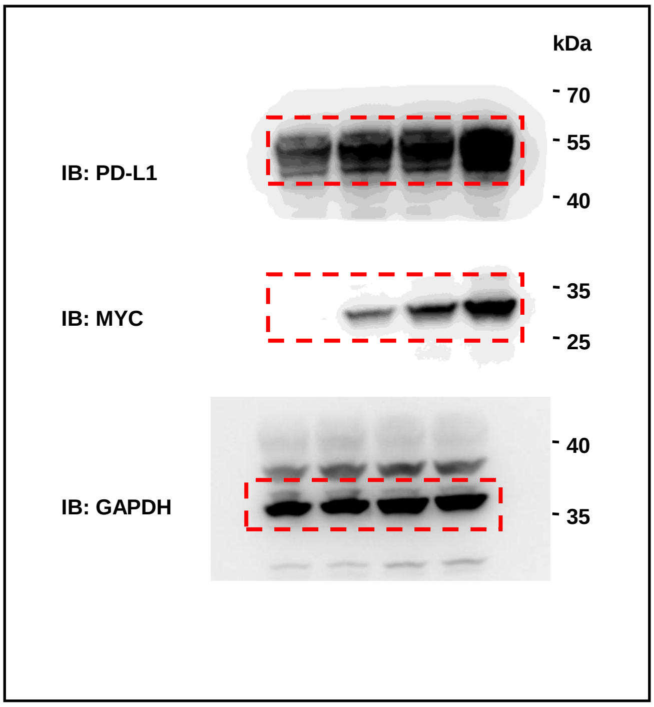

## Uncropped blots for Supplementary Fig. 11e

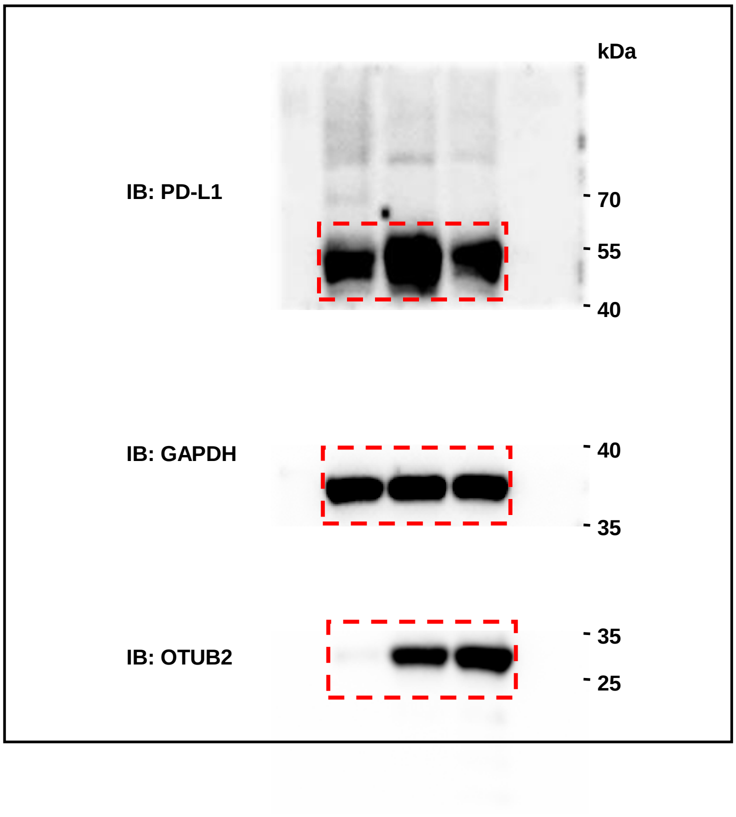


## Uncropped blots for Supplementary Fig. 11f

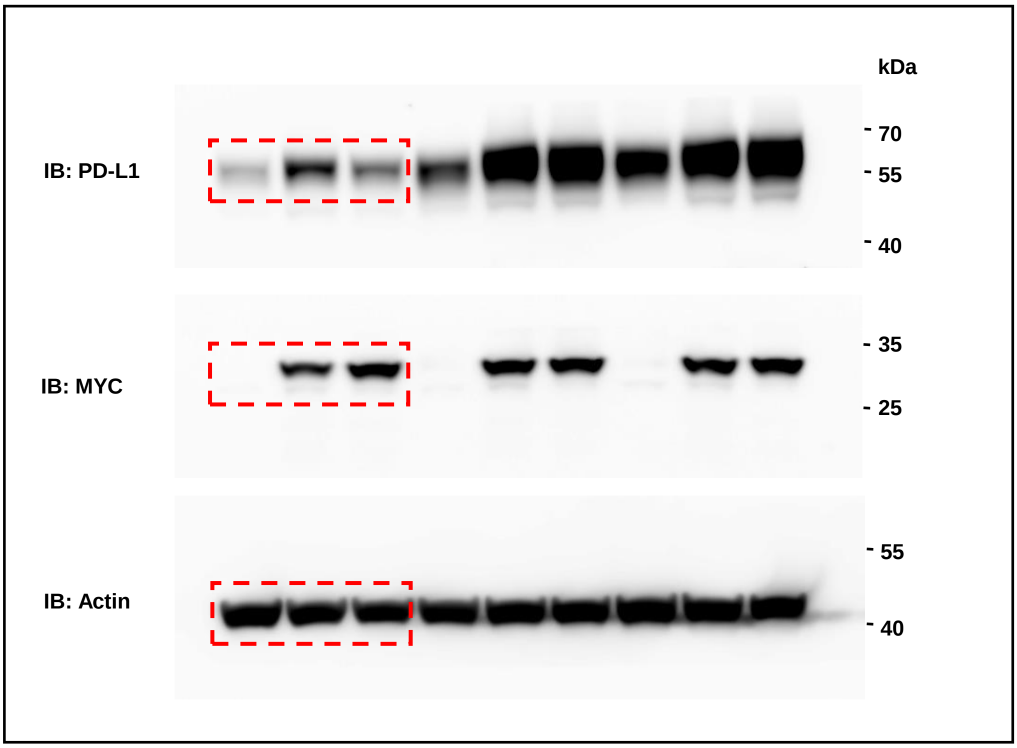

Uncropped blots for Supplementary Fig. 12a

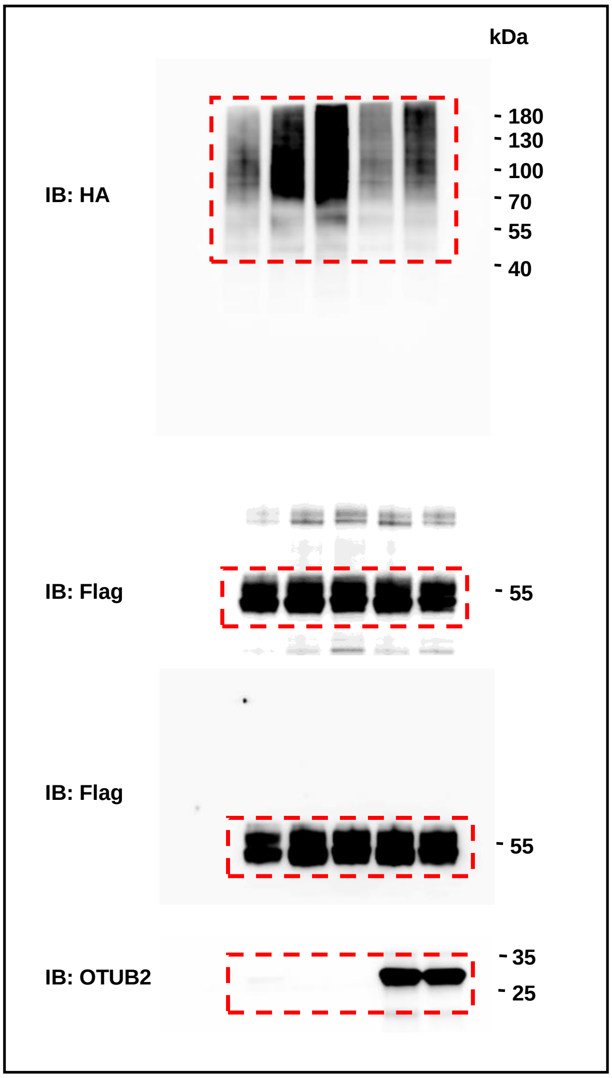


Uncropped blots for Supplementary Fig. 12b

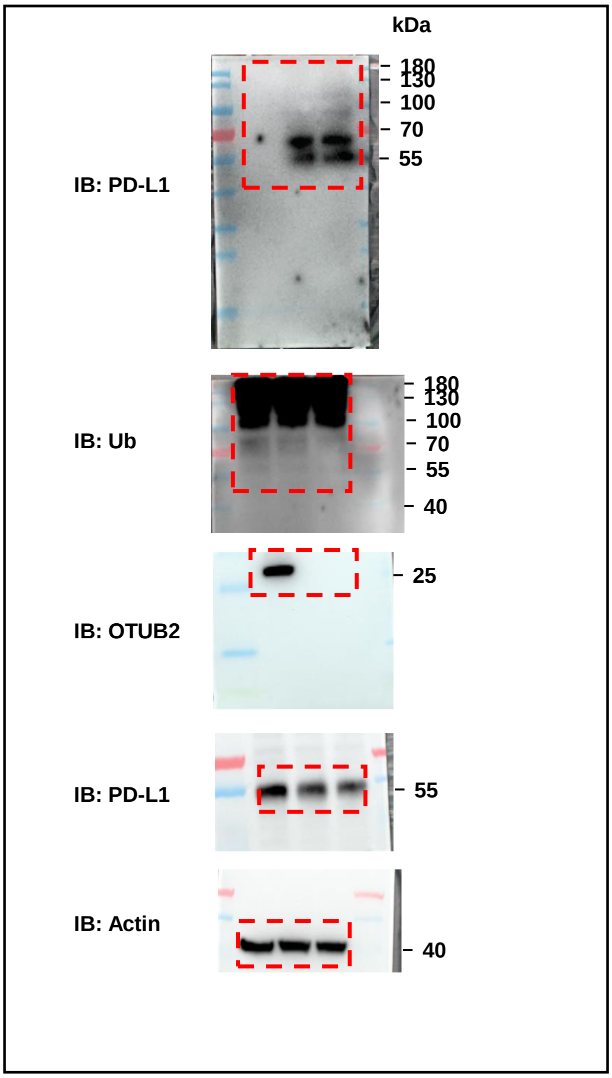

## Uncropped blots for Supplementary Fig. 13a

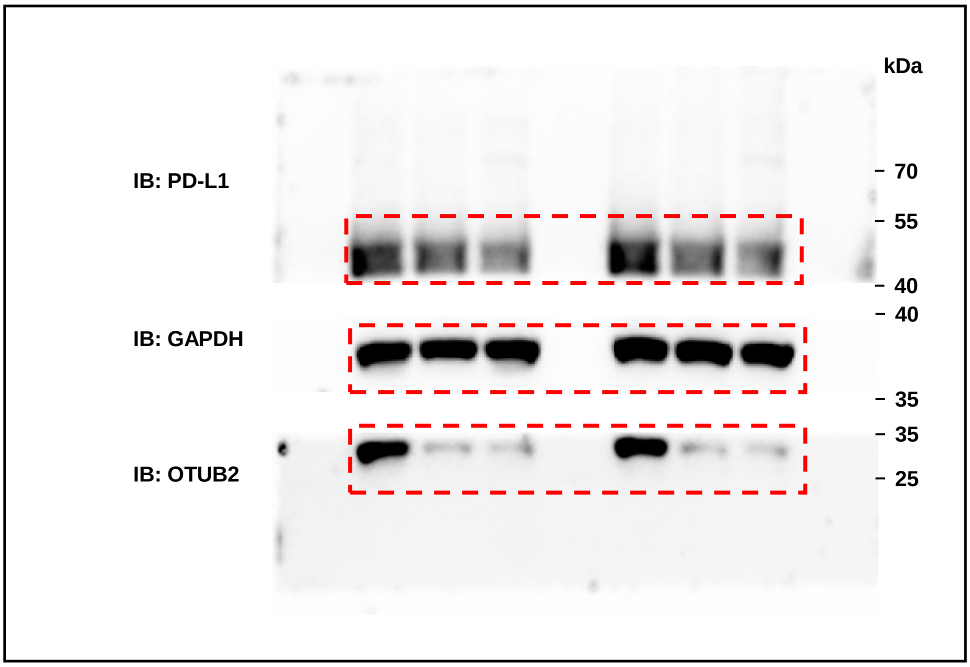


## Uncropped blots for Supplementary Fig. 13b

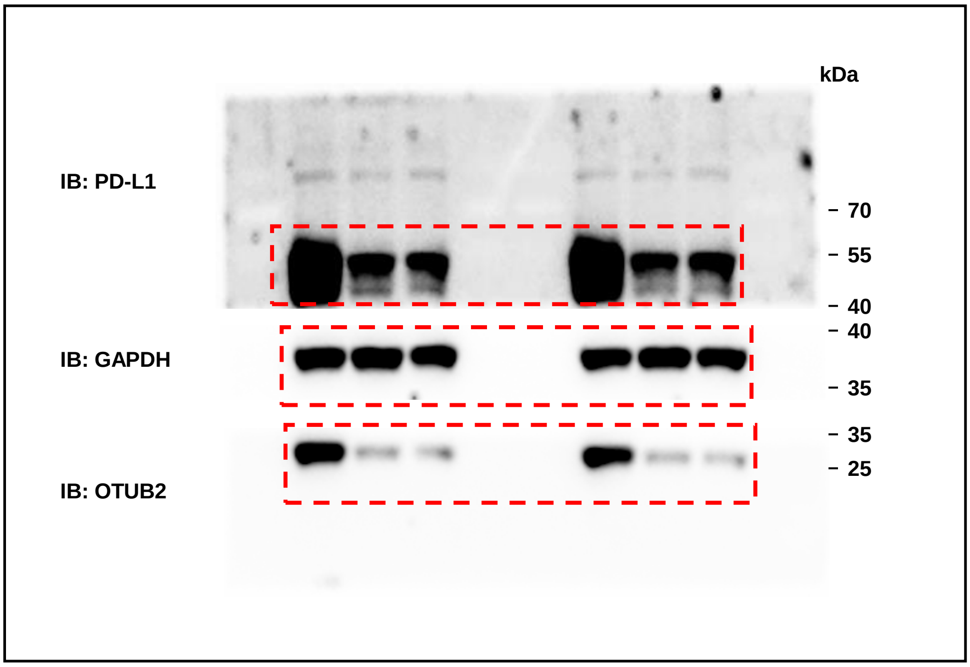

## Uncropped blots for Supplementary Fig. 15a

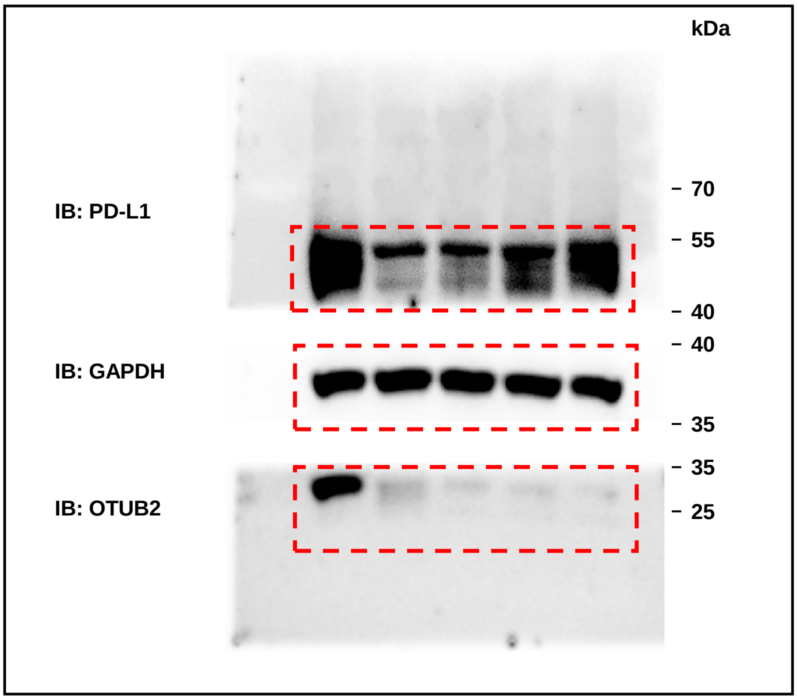


## Uncropped blots for Supplementary Fig. 15b

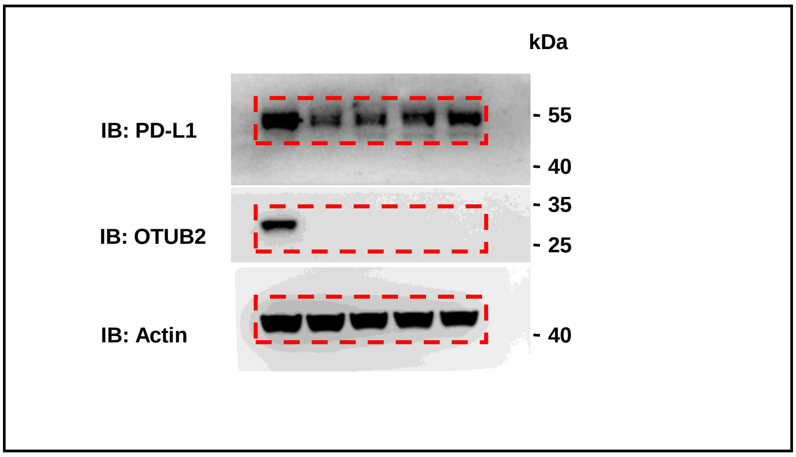


## Uncropped blots for Supplementary Fig. 15c

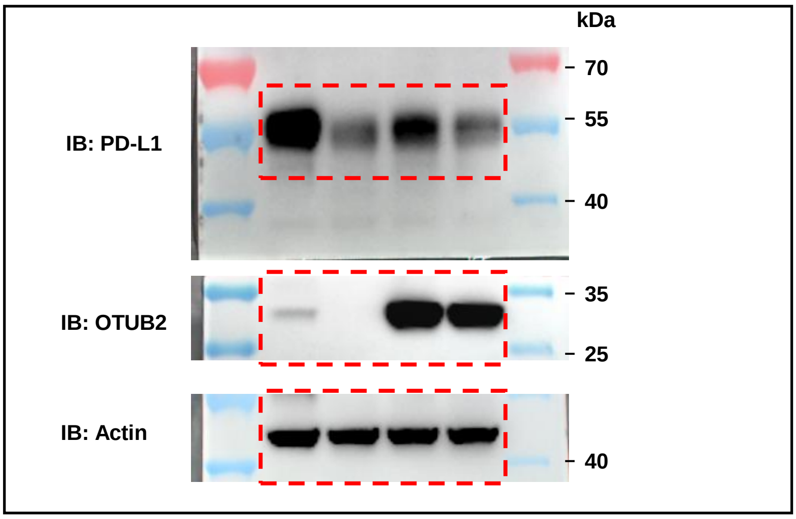

## Uncropped blots for Supplementary Fig. 19b

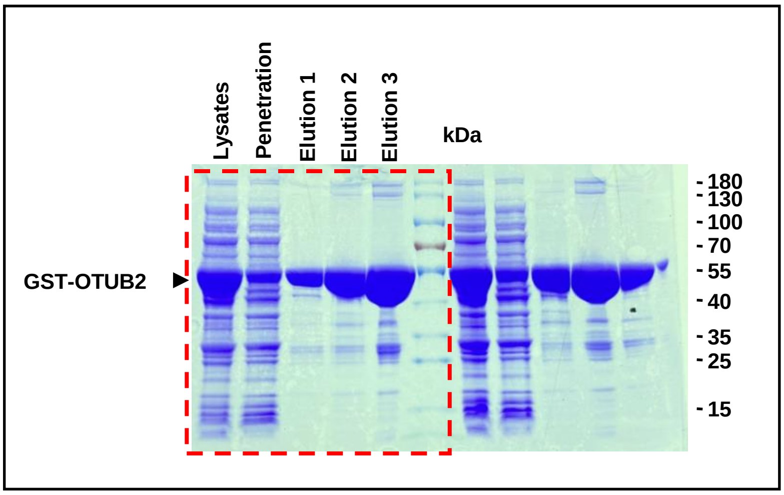


## Uncropped blots for Supplementary Fig. 19e

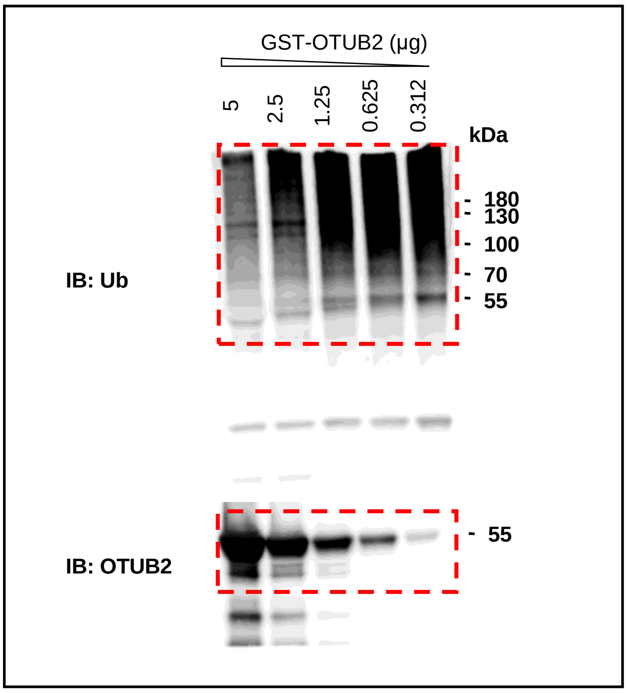

## Uncropped blots for Supplementary Fig. 20a

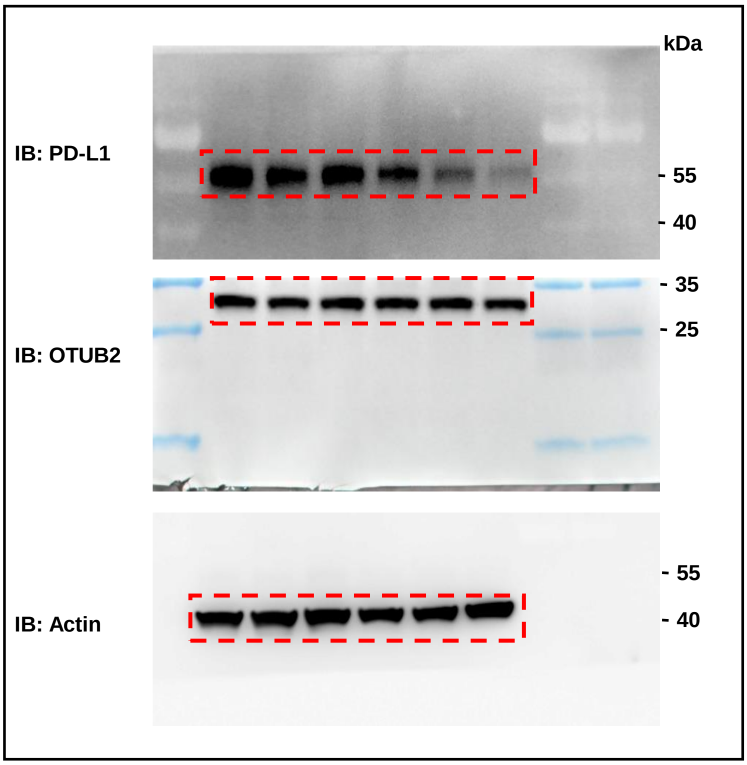


## Uncropped blots for Supplementary Fig. 20b

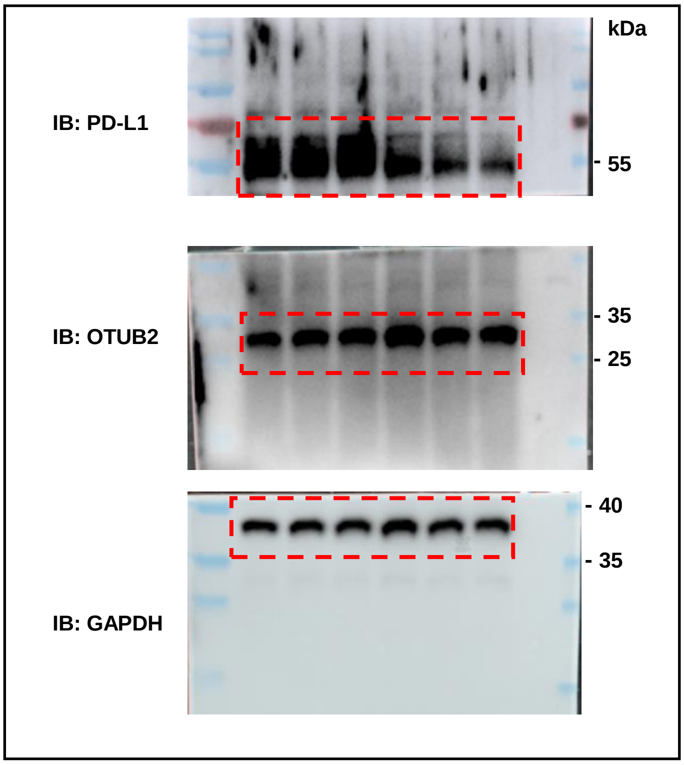


## Uncropped blots for Supplementary Fig. 20c

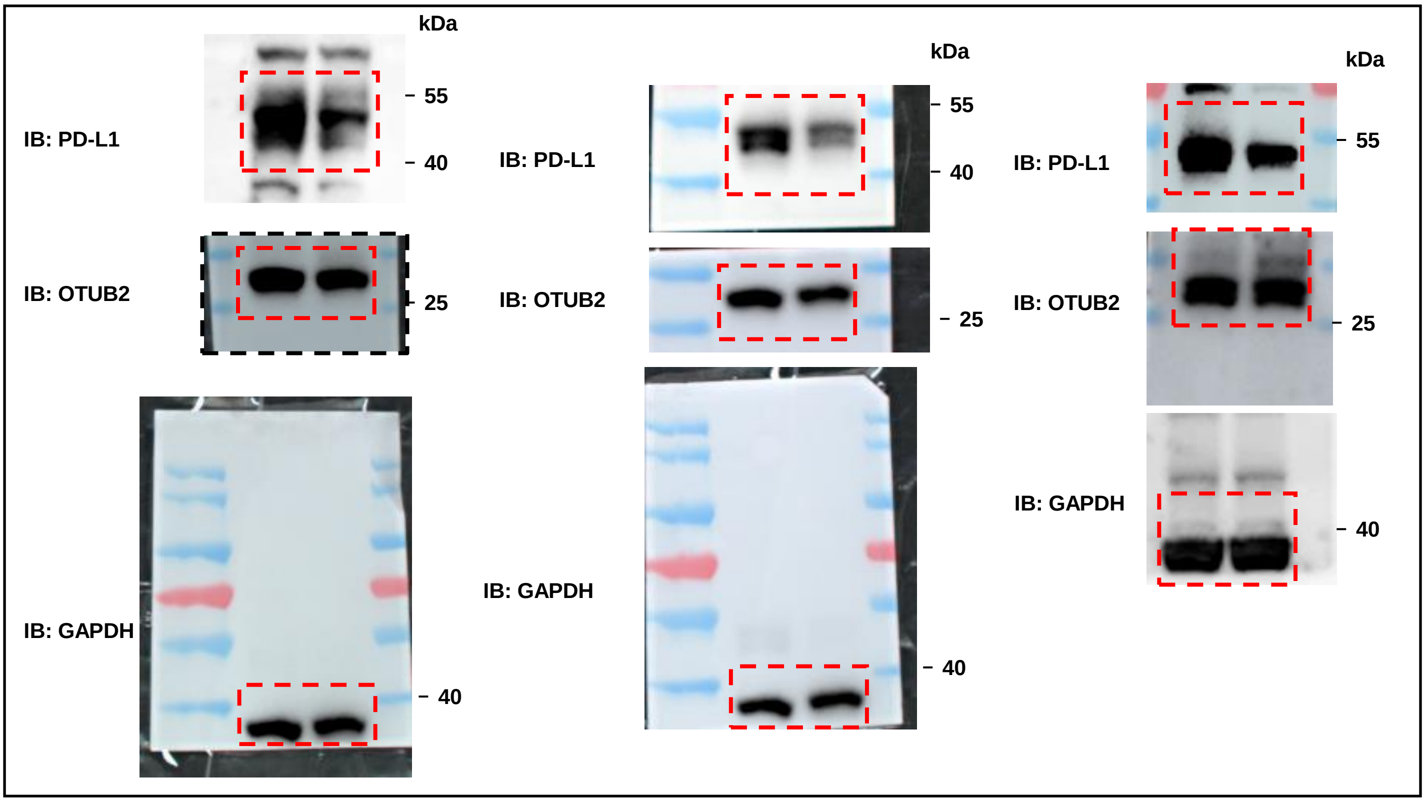

## Uncropped blots for Supplementary Fig. 21

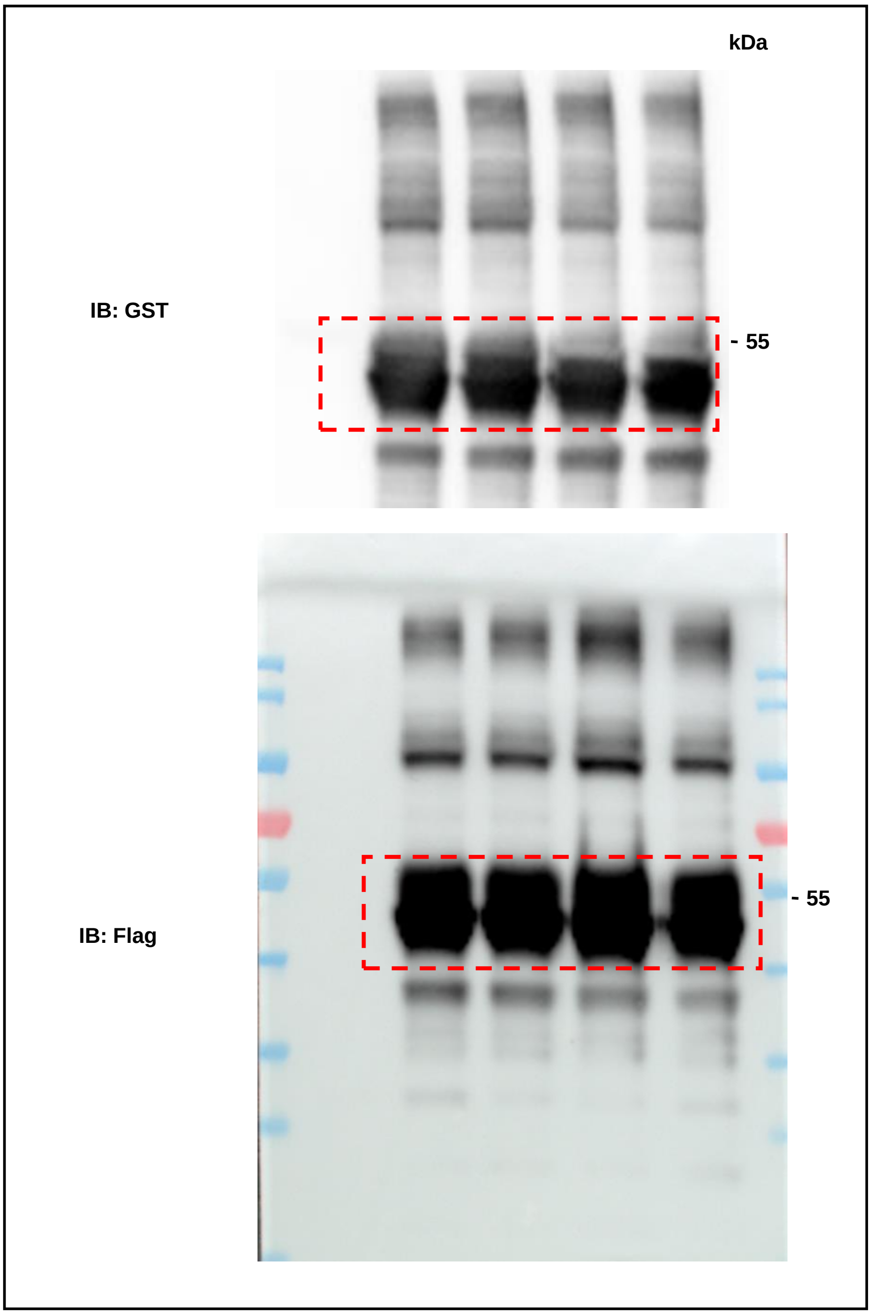

Supplement: Supplementary file 1 — Supplementary Information [file 41467_2023_44466_MOESM1_ESM.pdf]
