# Supplementary material for: Pharmaceutical targeting of OTUB2 sensitizes tumors to cytotoxic T cells via degradation of PD-L1
Source: Nat Commun. 2024 Jan 2;15:9. doi: 10.1038/s41467-023-44466-7 (PMC10761827; doi:10.1038/s41467-023-44466-7)
Supplement: Supplementary file 3 — Reporting Summary [file 41467_2023_44466_MOESM3_ESM.pdf]

nature portfolio

Corresponding author(s): Chenghao Huang, Pingguo Liu, Jun Zhang and Ningshao Xia

Last updated by author(s): Nov 22, 2023

Reporting Summary

Nature Portfolio wishes to improve the reproducibility of the work that we publish. This form provides structure for consistency and transparency in reporting. For further information on Nature Portfolio policies, see our Editorial Policies and the Editorial Policy Checklist.

Statistics

For all statistical analyses, confirm that the following items are present in the figure legend, table legend, main text, or Methods section.

n/a	Confirmed	
☐	☒	The exact sample size (*n*) for each experimental group/condition, given as a discrete number and unit of measurement
☐	☒	A statement on whether measurements were taken from distinct samples or whether the same sample was measured repeatedly
☐	☒	The statistical test(s) used AND whether they are one- or two-sided<br>*Only common tests should be described solely by name; describe more complex techniques in the Methods section.*
☒	☐	A description of all covariates tested
☐	☒	A description of any assumptions or corrections, such as tests of normality and adjustment for multiple comparisons
☐	☒	A full description of the statistical parameters including central tendency (e.g. means) or other basic estimates (e.g. regression coefficient) AND variation (e.g. standard deviation) or associated estimates of uncertainty (e.g. confidence intervals)
☐	☒	For null hypothesis testing, the test statistic (e.g. *F*, *t*, *r*) with confidence intervals, effect sizes, degrees of freedom and *P* value noted<br>*Give P values as exact values whenever suitable.*
☒	☐	For Bayesian analysis, information on the choice of priors and Markov chain Monte Carlo settings
☒	☐	For hierarchical and complex designs, identification of the appropriate level for tests and full reporting of outcomes
☐	☒	Estimates of effect sizes (e.g. Cohen's *d*, Pearson's *r*), indicating how they were calculated

Our web collection on statistics for biologists contains articles on many of the points above.

Software and code

Policy information about availability of computer code

Data collection	Flow cytometry data was recorded using FACSDIVA software (version 9.0)<br>Immunohistochemistry images were captured using Aperio ScanScope XT scanner (Leica)<br>IF images were captured using Leica Application Suite X (version 3.5.5.19976)<br>IHC images were captured using Leica Application Suite X (version 3.7.5.24914)<br>Western blotting data was recorded using Image Quant LAS 4000 min (version 1.3)
Data analysis	Flow cytometry was analyzed using FlowJo (version 10.0.7r2)<br>Virtual screening was analyzed using Schrödinger (Maestro version 11.4)<br>Data was visualized using GraphPad Prism software (version 7.0)<br>Images were analyzed with ImageJ (version 1.53t)<br>Surface Plasmon Resonance (SPR) data were analyzed using Biacore Insight Evaluation Software (version 3.0.11.15423)<br>TIMER2.0 http://timer.cistrome.org/<br>GEPIA http://gepia.cancer-pku.cn/index.html<br>Phantasus v1.5.1 https://genome.ifmo.ru/phantasus

For manuscripts utilizing custom algorithms or software that are central to the research but not yet described in published literature, software must be made available to editors and reviewers. We strongly encourage code deposition in a community repository (e.g. GitHub). See the Nature Portfolio guidelines for submitting code & software for further information.

Data

Policy information about availability of data

All manuscripts must include a data availability statement. This statement should provide the following information, where applicable:

- Accession codes, unique identifiers, or web links for publicly available datasets
- A description of any restrictions on data availability
- For clinical datasets or third party data, please ensure that the statement adheres to our policy

The publicly available human LUSC, LUAD and STAD RNA-seq data used in this study are available on the UCSC Xena Browser (https://xenabrowser.net/). The publicly available TCGA RNA-seq data used in this study are available through the TIMER2.0 (http://timer.cistrome.org/) and GEPIA (http://gepia.cancer-pku.cn/index.html). The remaining data are available within the Article, Supplementary Information or Source Data file.

Human research participants

Policy information about studies involving human research participants and Sex and Gender in Research.

Reporting on sex and gender	LUSC cohort: median age at resection was 63.5 years, average 63.8 years (min-max: 36-78 years). 84 patients were male and 6 patients were female; LUAD cohort: median age at resection was 63.0 years, average 63.0 years (min-max: 35-86 years). 51 patients were male and 41 patients were female.
Population characteristics	A human LUSC microarray (HLugS180Su01, 90 cancer tissues from patients, and 90 adjacent paracancerous tissues with IHC staining data for CD8 and PD-L1) or a human LUAD microarray (HLugA180Su03, 92 cancer tissues from patients, and 88 adjacent paracancerous tissues with IHC staining data for PD-L1) was purchased from Shang Hai Outdo Biotech Company (https://www.superchip.com.cn/biology/tissue.html).
Recruitment	Written informed consent was obtained from all patients prior to sample collection in accordance with the Declaration of Helsinki. Patients were randomly recruited without self-selection bias.
Ethics oversight	All procedures performed in studies involving human participants were in accordance with the ethical standards of the institutional ethics committee (no. SHYJS-CP-1810010 and SHYJS-CP-1610001) and with the Helsinki declaration.

Note that full information on the approval of the study protocol must also be provided in the manuscript.

Field-specific reporting

Please select the one below that is the best fit for your research. If you are not sure, read the appropriate sections before making your selection.

☒ Life sciences ☐ Behavioural & social sciences ☐ Ecological, evolutionary & environmental sciences

For a reference copy of the document with all sections, see nature.com/documents/nr-reporting-summary-flat.pdf

Life sciences study design

All studies must disclose on these points even when the disclosure is negative.

Sample size	Sample sizes were estimated based on previous experience and preliminary analyses, and are determined by power analyses to be the minimum number needed to achieve statistical significance for the work proposed (G*Power, Faul et al. 2007, where power is 90% and p value < 0.05).
Data exclusions	In murine models, tumors with ulcerations were excluded. After IHC staining, the tissue sections detached from slides were excluded.
Replication	All findings were replicated in two to three separate experiments (as described in detail in the Methods and Figure Legends section). All separate experiments yielded comparable trends and results.
Randomization	In all experiments, grouping was performed randomly. For implantations of OTUB2-KO or control tumor cells, mice were earmarked before grouping and randomly separated into groups. For in vivo studies evaluating the efficacy of small-molecule compounds, experimental mice were randomized according to tumor volume to ensure equal mean tumor volumes per condition at the start of treatment.
Blinding	For immunohistochemistry analysis, tissue sections were de-identified and blinded for pathological analysis of histochemical scores. For histochemical analysis of mouse tumor tissues, pathologist was blinded to group of mice for analysis. The investigators were not blinded during outcome assessment for other experiments since data acquisition and analysis were done using indicated software.

Reporting for specific materials, systems and methods

We require information from authors about some types of materials, experimental systems and methods used in many studies. Here, indicate whether each material, system or method listed is relevant to your study. If you are not sure if a list item applies to your research, read the appropriate section before selecting a response.

Materials & experimental systems

n/a	Involved in the study
☐	☒ Antibodies
☐	☒ Eukaryotic cell lines
☒	☐ Palaeontology and archaeology
☐	☒ Animals and other organisms
☒	☐ Clinical data
☒	☐ Dual use research of concern

Methods

n/a	Involved in the study
☒	☐ ChIP-seq
☐	☒ Flow cytometry
☒	☐ MRI-based neuroimaging

Antibodies

Antibodies used

Antibodies, Supplier, Catalog number, Clone name, Dilution
Brilliant Violet 605 anti-mouse CD45 Antibody, BioLegend, 103140, 30-F11, 1:200
FITC anti-mouse CD8a Antibody, BioLegend, 100706, 53-6.7, 1:200
APC anti-mouse IFN-γ Antibody, BioLegend, 505810, XMG1.2, 1:200
PE/Cyanine7 anti-human/mouse Granzyme B Recombinant Antibody, BioLegend, 372214, QA16A02, 1:200
APC anti-mouse CD274 (B7-H1, PD-L1) Antibody, BioLegend, 124312, 10F.9G2, 1:200
APC anti-human CD274 (B7-H1, PD-L1) Antibody, BioLegend, 329708, 29E.2A3, 1:200
Anti-OTUB2 mouse monoclonal antibody, Sangon Biotech, D199590, 1:500
Anti-PD-L1 antibody, Abcam, ab213480, EPR20529, 1:500
PD-L1/CD274 Monoclonal antibody, ProteinTech, 66248-1-Ig, 2B11D11, 1:500
Beta Actin Monoclonal antibody, ProteinTech, 66009-1-Ig, 2D4H5, 1:2,000
GAPDH Monoclonal antibody, ProteinTech, 60004-1-Ig, 1E6D9, 1:2,000
Ubiquitin (P4D1) Mouse mAb, CST, 3936, 1:1,000
Anti-HA antibody, Mouse monoclonal, Sigma–Aldrich, H3663, HA-7, 1:2,000
Monoclonal ANTI-FLAG® M2 antibody, Sigma–Aldrich, F1804, M2, 1:2,000
Anti-c-Myc antibody, Sigma–Aldrich, C3956, polyclonal, 1:500
Goat Anti-Mouse IgG (H + L)-HRP Conjugate, Bio-Rad, 1706516, polyclonal, 1:3,000
Goat Anti-Rabbit IgG (H + L)-HRP Conjugate, Bio-Rad, 1706515, polyclonal, 1:3,000
InVivoMAb anti-mouse CD8α, Bio X Cell, BE0061, 2.43, 200 μg/mouse
InVivoMAb rat IgG2b isotype control, Bio X Cell, BE0090, LTF-2, 200 μg/mouse
OTUB2 Antibody, Affinity, AF9147, polyclonal, 1:400
Calnexin (C5C9) Rabbit mAb, CST, 2679S, 1:400
Donkey anti-Mouse IgG (H+L) Highly Cross-Adsorbed Secondary Antibody, Alexa Fluor 488, Invitrogen, A-21202, polyclonal, 1:2,000
Anti-Rabbit IgG (whole molecule)–TRITC antibody, Sigma–Aldrich, T6778, polyclonal, 1:500
Donkey anti-Rabbit IgG (H+L) Highly Cross-Adsorbed Secondary Antibody, Alexa Fluor 488, Invitrogen, A-21206, polyclonal, 1:2,000
F(ab')2-Goat anti-Mouse IgG (H+L) Cross-Adsorbed Secondary Antibody, Alexa Fluor 647, Invitrogen, A-21237, polyclonal, 1:500
Anti-CD8 alpha antibody, Abcam, ab209775, EPR20305, 1:1,000
Anti-Granzyme B antibody, Abcam, ab4059, polyclonal, 1:100
PD-L1/CD274 Polyclonal antibody, ProteinTech, 17952-1-AP, polyclonal, 1:1,000
Anti-OTUB2 antibody, Abcam, ab175200, EPR13028(B), 1:200
Anti-USP22 antibody, Abcam, ab195289, EPR18945, 1:200
Anti-CSN5 antibody, CST, 6895S, polyclonal, 1:200
Anti-USP7 antibody, CST, 4833T, D17C6, 1:2,000
Anti-YAP antibody, Affinity, DF3182, polyclonal, 1:100
Anti-Phospho-AKT1 (S473) antibody, Abcam, ab81283, EP2109Y, 1:100
Anti-Phospho-p65 (S536) antibody, Affinity, AF2006, polyclonal, 1:100
Anti-GST-HRP antibody, ProteinTech, HRP-66001, 3G12B10, 1:500
Anti-GST antibody, Sino Biological, 11213-RP01, polyclonal, 1:2,000
Goat Anti-Rabbit IgG (H + L)-HRP Conjugate, Bio-Rad, 1706515, polyclonal, 1:3,000
Goat Anti-Mouse IgG (H + L)-HRP Conjugate, Bio-Rad, 1706516, polyclonal, 1:3,000

Validation

All antibodies were obtained from commercial vendors and specificity was based on the descriptions and information provided by the Manufacturers. For the OTUB2 antibody (D199590), an internal knock out validation was successfully conducted, which was shown in Supplementary Fig. 6a (cells with and without OTUB2 KO by CRIPSR/Cas9 technology were stained and Cells with OTUB2 KO had confirmed no staining with this antibody).

Eukaryotic cell lines

Policy information about cell lines and Sex and Gender in Research

Cell line source(s)	B16-F10, LL/2, CT26.WT, NCI-H358, LoVo, SK-MES-1, NCI-H226, KLN205, MDA-MB-231, NCI-1975, FaDu, BT-549, U-87 MG, Hep-2, HeLa, BT-20, HCC1937 and 293T cells were purchased from American Type Culture Collection (ATCC, Manassas, VA, USA). MC38 cells were purchased from the National Infrastructure of Cell Line Resources (Beijing, China).
Authentication	Cell lines were freshly ordered from reliable sources with authentication certification (ATCC) for B16-F10, LL/2, CT26.WT, NCI-H358, LoVo, SK-MES-1, NCI-H226, KLN205, MDA-MB-231, NCI-1975, FaDu, BT-549, U-87 MG, Hep-2, HeLa, BT-20, HCC1937 and 293T cells. MC38 cells were obtained from reliable academic sources (National Infrastructure of Cell Line Resources). No other authentication was performed.
Mycoplasma contamination	Parental lines and their derivatives were confirmed to be mycoplasma negative using the LookOut® Mycoplasma PCR Detection Kit (Sigma–Aldrich, MP0035)
Commonly misidentified lines (See ICLAC register)	None

Animals and other research organisms

Policy information about studies involving animals; ARRIVE guidelines recommended for reporting animal research, and Sex and Gender in Research

Laboratory animals	Species: mus musculus<br>Strains: C57BL/6J, C57BL/6-Tg(TcraTcrb)1100Mjb/J (OT-I), NOD-SCID, DBA/2<br>Sex: female<br>Age: 5-6 weeks<br>All mice were maintained in a specific-pathogen-free (SPF) facility and were housed at a temperature of 25 °C in a humidity-controlled environment with free access to food and water in a 12h light/dark cycle.
Wild animals	Wild animals were not used in this study
Reporting on sex	Female mice were used in experiments.
Field-collected samples	None
Ethics oversight	All work in this study was approved by the Institutional Animal Care and Use Committee of Xiamen University (XMULAC20190072).

Note that full information on the approval of the study protocol must also be provided in the manuscript.

Flow Cytometry

Plots

Confirm that:

☒ The axis labels state the marker and fluorochrome used (e.g. CD4-FITC).

☒ The axis scales are clearly visible. Include numbers along axes only for bottom left plot of group (a 'group' is an analysis of identical markers).

☒ All plots are contour plots with outliers or pseudocolor plots.

☒ A numerical value for number of cells or percentage (with statistics) is provided.

Methodology

Sample preparation	See Method section of our manuscript.<br>To analyze in vitro cell viability by flow cytometry analysis, cells were trypsinized and analyzed with the Zombie Aqua™ Fixable Viability Kit (BioLegend, 423102) 120 h after plating. For analysis of Strawberry positive cells, cells were trypsinized and analyzed by flow cytometry analysis 24 h after plating. For PD-L1 analysis, 48 h after plating, cells were trypsinized and stained with anti-PD-L1-APC for 20 min on ice.<br>Mouse tumors were dissected and dissociated with 1 mg/ml Collagenase D (Roche) and 100 μg/ml DNase I (Sigma) in RPMI-1640 medium supplemented with 2% FBS for 1.5 h with continuous agitation. The digestion mixture was homogenized by repeated pipetting and filtered through a 70-μm nylon filter. The single cell suspensions were washed twice with a flow cytometry staining buffer and stained with the Zombie Aqua™ Fixable Viability Kit to eliminate dead cells. For surface staining, after washing twice, the cells were stained with the corresponding antibodies, incubated for 30 min at 4°C, and then subjected to flow cytometry analysis. For intracellular staining, cells were fixed and permeabilized using Fixation/ Permeabilization Solution Kit (BD, 554714). Intracelluar antibodies were then stained for 30 min on ice. After washing twice, the cells were then subjected to flow cytometry analysis.
Instrument	Immunostained cells were subsequently subjected to flow cytometry analysis by using a BD LSRFortessa™ X-20 flow

Instrument	cytometer.
Software	Cell profiles were first recorded using FACSDIVA software (version 9.0), and the data were then analyzed using FlowJo (version 10.0.7r2).
Cell population abundance	None
Gating strategy	Cultured tumor cells: morphology (FSC/SSC) -> singe cells (FSC-A/FSC-H)-> live cells (BV510-) -> Strawberry+ or PD-L1+ cells.<br>Primary bulk tumor cells: morphology (FSC/SSC) -> single cells (FSC-A/FSC-H) -> live cells (BV510-) -> CD45+ -> CD8+ -> IFN-γ+ or GZMB+ cells.<br>Boundaries between "positive" and "negative" populations were defined using a fluorescence minus one sample.

☒ Tick this box to confirm that a figure exemplifying the gating strategy is provided in the Supplementary Information.
